# The immunological landscape of the area postrema in neuromyelitis optica spectrum disorders

**DOI:** 10.1111/bpa.70127

**Published:** 2026-07-26

**Authors:** Qian Yu, Yoshiki Takai, Naoya Yamazaki, Sarah Brandl, Katharina M. Mair, Thibault Bouderlique, Maria Eleni Kastriti, Igor Adameyko, Romana Höftberger, Markus Reindl, Jan Bauer, Monika Bradl

**Affiliations:** ^1^ Center for Brain Research, Division of Neuroimmunology Medical University of Vienna Vienna Austria; ^2^ Department of Neurology Tohoku University Graduate School of Medicine Sendai Japan; ^3^ Clinical Department of Neurology Medical University of Innsbruck Innsbruck Austria; ^4^ Division of Neuropathology and Neurochemistry, Department of Neurology Medical University of Vienna Vienna Austria

**Keywords:** animal model, area postrema, chemokines, microglia, neuromyelitis optica, retinoic acid

## Abstract

Patients suffering from aquaporin‐4 antibody‐positive neuromyelitis spectrum disorder may present with radically different types of lesions in the central nervous system. They may show large tissue destructive lesions, as typically seen in medulla, spinal cord, and optic nerves, and lesions with much better tissue preservation, as seen in the area postrema. The causes for the resilience of the area postrema to the action of aquaporin 4‐antibodies are currently unclear. We performed detailed immunohistochemical analysis combined with spatial transcriptomics and functional annotation clustering, and identified key differences in the immunological landscape between the area postrema and the medulla. In the area postrema, only few chemokines were upregulated in response to AQP4 antibodies. Therefore, the immune response was dominated by local microglial/macrophages which displayed a low grade of microgliosis and an overall low level of activation. Il‐1β expression was below our cut‐off for detection. This was in marked contrast to lesions in the medulla, which were characterized by the expression of many chemokines underlying the recruitment of macrophages and neutrophils from the periphery to this site, and by Il‐1β expression. Aldehyde dehydrogenase 1 family member A2 (Aldh1a2), the key molecule for retinoic acid synthesis, was strongly expressed in the area postrema, but not in the medulla. Because retinoic acid can prevent the activation of astrocytes and microglia, can reduce the production of cytokine/chemokine transcripts, can alleviate the expression of IL‐1β, and can promote an M1‐ to M2‐transition in macrophages, our data implicate a protective role of retinoic acid at this site.

## Graphical abstract

Aquaporin‐4 antibody‐positive neuromyelitis spectrum disorder is characterized by large tissue destructive lesions in medulla, spinal cord, and optic nerves, with only partial recovery from clinical symptoms, and by lesions with very little tissue destruction and mostly complete recovery from clinical symptoms, as seen in the area postrema. Immunohistochemical and spatial transcriptomic analysis revealed a dampened innate immune response in the area postrema, correlating with the strong expression of aldehyde dehydrogenase 1 family member a2 at this site.

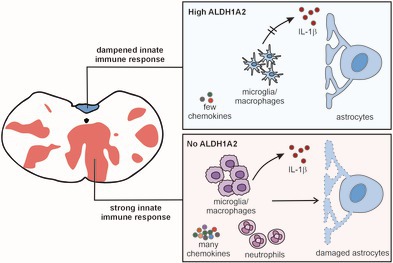

## 1 INTRODUCTION

Typically, neuromyelitis optica spectrum disorder (NMOSD) is described as an autoimmune disease associated with the presence of pathogenic aquaporin‐4 (AQP4)‐specific autoantibodies (abs) in the vast majority of patients, and with the presence of large tissue destructive lesions in optic nerves and spinal cord. At these sites, lesions are initiated when the AQP4‐abs cross through the blood–brain barrier (BBB), bind to AQP4 on the surface of astrocytic endfeet, and lead to the destruction of these cells by complement‐dependent cytotoxicity (CDC) and/or antibody‐dependent cellular cytotoxicity (ADCC) [1, 2], often with residual disability for vision or motility. However, there is a third clinical manifestation of NMOSD: area postrema syndrome (APS), characterized by intractable hiccups, nausea, and vomiting. The corresponding lesions in the area postrema are characterized by loss of AQP4 immunoreactivity and inflammation, but the resulting tissue damage at this site is much less severe than seen elsewhere in the central nervous system (CNS) of NMOSD patients, essentially resolves, and allows for affected patients to remit from the associated symptoms [3]. Why is the area postrema so resilient to pathogenic AQP4‐abs?

Here, we addressed this question in our AQP4‐antibody mediated animal model of NMOSD and took advantage of spatial transcriptomics, functional annotation clustering of identified transcripts, and immunohistochemical analysis to characterize the response of the area postrema to AQP4‐abs challenge over time, and to reveal profound differences to AQP4‐abs induced lesions in the medulla.

## 2 MATERIALS AND METHODS

### 2.1 NMOSD patients in long‐term follow‐up and MRI study

We reviewed medical records of patients who visited the Department of Neurology, Tohoku University Hospital, from January 2000 to December 2023. A total of 151 patients with AQP4‐abs+ NMOSD who fulfilled the criteria of the International Panel for NMO Diagnosis (IPND) [4] were identified, but 42 of them were excluded for the following reasons: second opinion (*n* = 18), follow‐up less than a year (*n* = 13), insufficient information (*n* = 9), and use of rituximab for other diseases (*n* = 2). From the remaining 109 patients (with 464 attacks of NMOSD), 39 female patients were available for our study, with altogether 71 attacks with APS (for further patient information, see Table 1). All patients provided informed consent to the use of their medical records for research purposes. The study was approved by the ethical committee of Tohoku University School of Medicine (Nr. 2022‐1‐1103).

**TABLE 1:** Clinical information about 39 female NMOSD patients with a total number of 71 attacks of area postrema syndrome (APS) in long‐term follow up and MRI.

Baseline characteristics	Information on APS attacks
Age at attack	44 ± 14 years
Coexisting attack	
Brain lesion	6/71 (8.5%)
Myelitis	24/71 (34%)
Optic neuritis	11/71 (15%)
Isolated AP symptoms	40/71 (56%)
APS symptoms	
Hiccup	49/71 (69%)
Nausea	40/71 (56%)
Vomit	20/71 (28%)
Cough	3/71 (4.2%)
Acute AP lesion confirmed by MRI	35/52 (67%)
Residual AP symptoms after acute phase	3/58 (5.2%)

### 2.2 NMOSD patients in autopsy study

We analyzed tissue specimens from 18 autopsied patients with NMOSD (for further patient information, see Table 2) by immunohistochemistry. Ten cases were analyzed at Tohoku University Hospital, and the remaining eight cases were analyzed at Aichi Medical University. In 12 cases, AQP4‐IgG seropositivity had been confirmed by cell‐based assay (CBA) during the patients' lifetime. The remaining six cases showed clinical features consistent with NMOSD and pathological findings characteristic of astrocytopathy associated with NMOSD [5]. This part of our study was approved by the ethical committee of Tohoku University School of Medicine (No. 2023‐11144), and by the Ethics Committees of the Medical University of Vienna (EK‐No. 1636/2019 and 1123/2015/Version 25). For comparison with the NMOSD cases, we also examined ALDH1A2 expression in the area postrema of seven cases with non‐neurological causes of death, one case with ALS, and three cases with multiple sclerosis (MS).

**TABLE 2:** Clinicopathological characteristics of autopsied NMOSD cases and analyzed CNS lesions.

Case No.	Age	Sex	Disease duration, month	Duration after last attack, month	AQP4‐Ab	Stage of the lesions	APL	BSL	SCL
1	23	F	148	3	NA	Acute–chronic	+	+	+
2	53	F	3	2	Positive	Subacute	+	+	+
3			44	4	NA	Subacute–chronic	+	+	+
4	66	F	120	36	Positive	Chronic	+	−	+
5	56	F	228	108	Positive	Chronic	+	−	+
6	71	F	252	22	Positive	Chronic	+	−	+
7	73	F	204	50	NA	Chronic	+	−	+
8	78	F	2	0.4	Positive	Acute–subacute	NA	+	+
9	23	F	240	0.5	NA	Acute–chronic	NA	+	+
10	63	M	0.6	0.6	Positive	Subacute	NA	+	+
11	57	M	8	0.5	Positive	Subacute	NA	+	+
12	46	F	84	0.6	NA	Subacute–chronic	NA	+	+
13	48	M	297	NA	Positive	Chronic	−	+	+
14	87	M	20	15	Positive	Chronic	−	+	+
15	64	F	276	48	Positive	Chronic	NA	+	+
16	74	F	2	2	NA	Subacute–chronic	−	−	+
17	46	F	252	12	Positive	Chronic	NA	−	+
18	46	F	156	108	Positive	Chronic	NA	−	+

Abbreviations: Ab, antibody; APL, area postrema lesion; AQP4, aquaporin 4; BSL, brain stem lesion; F, female; M, male; NA, not applicable; Pt, patient; SCL, spinal cord lesion; +, lesion available for analysis; −, lesion not available for analysis.

### 2.3 Animals and tissue samples

Seven weeks old Lewis rats were obtained from Charles River Wiga (Sulzfeld, Germany). For all experiments, a single rat was considered as a biological *n*. We only used female rats, because NMOSD preferentially affects female persons, and because male and female rats significantly differ in weight at this age (female rats on average 150 g, male rats on average 170 g). Using both sexes in the experiments would have introduced unwanted noise into the final pathological assessment, and would therefore have led to an unnecessary increase in the numbers of animals, which would not have complied with the 3R policy about the usage of animals in research. For 1 week prior to, and throughout the experiments, the animals were housed in the Decentral Facilities of the Institute for Biomedical Research (Medical University Vienna) under standardized conditions. All applicable international, national, and/or institutional guidelines for the care and use of animals were followed, and the in vivo studies are reported in accordance with the ARRIVE guidelines for reporting experiments involving animals (https://arriveguidelines.org/arrive-guidelines).

All procedures performed in the animals were in accordance with the ethical standards of the Medical University of Vienna, and were approved by the Ethic Commission of the Medical University Vienna and performed with the license of the Austrian Ministry for Science and Research (GZ: BMBWF‐66.009/0107‐V/3b/2018).

Experimental groups consisted of 14 animals which have been daily injected intraperitoneally (i.p.) under isoflurane anesthesia for 1 (*n* = 4), 2 (*n* = 5), and 4 consecutive days (*n* = 5) with 1 mg of the monoclonal AQP4‐specific antibody E5415A [6, 7] (isolated from the hybridoma cell line AQP4 [E5415A‐1H6‐68, Resource no. RCB4883, provided by the Riken BRC through the National BioResource Project of the MEXT/AMED, Japan], termed “mAQP4‐abs” throughout the manuscript) in a concentration of 1 mg/mL in phosphate‐buffered saline (PBS). Twenty‐four hour after the last injection, the animals were killed with CO_2_ inhalation for immunohistochemical evaluations. We did not use blinding and also did not randomize the groups because we knew from our previous work that pathological changes in the area postrema cannot be detected in the living animal [8], because rats cannot vomit [9] and thus do not show typical signs of APS. We have chosen this protocol to create experimental groups, because it reproducibly generates animals with pathological changes in the area postrema within a time window of 1–5 days after the first i.p. application of E5415A, with differences in the timing of onset between individual animals [8]. We also knew that the pathological changes fall into two different categories: an earlier stage clearly defined by a patchy loss of AQP4 reactivity, and a later stage with complete loss of AQP4 in the area postrema [8], which allows us to study both early and late effects at this site. This is the best approximation to the human AQP4‐ab challenged area postrema we could make, because NMOSD patients do not die from an isolated attack of APS, but die as a result of very severe inflammatory lesions elsewhere in the CNS, and might have, at the time of death, an involvement of the area postrema as well.

We further used archival tissue from our previous paper [8] to have at least 8 animals per experimental group, as suggested by statistical sample size analysis prior to the experiments. So, in total we had CNS tissue of the following new (N) and archival (A) age‐matched samples available:

- Control group which encompassed archival tissue of 17 female Lewis rats which were either untreated (*n* = 4A), or had been intraperitoneally injected daily for 1 (*n* = 4A), 2 (*n* = 5A), or 5 consecutive days (*n* = 4A) with control mouse IgG (Sigma, Vienna, Austria), in a concentration of 1 mg/mL in PBS [8].
- Animals with patchy loss of AQP4 reactivity (*n* = 5N + 3A).
- Animals with complete loss of AQP4 reactivity in the area postrema (*n* = 9N).

Our spatial transcriptomics study was exploratory, and used four rats in total. The animals were left untreated (*n* = 1), or were intraperitoneally injected daily for 1 (*n* = 1), 2 (*n* = 1), or 5 consecutive days (*n* = 1) with mAQP4‐abs, and killed by CO_2_ inhalation 24 h after the last injection. From this regimen, we obtained one intact area postrema, one area postrema with patchy loss of AQP4 reactivity, and one area postrema with complete loss of AQP4 reactivity.

### 2.4 Immunohistochemical stainings of paraffin‐embedded tissue

Human tissue: Four to five micrometer‐thick sections of paraffin‐embedded tissues were evaluated by immunohistochemical techniques. Briefly, paraffin‐embedded sections were deparaffinized in xylene and rehydrated through graded ethanol. After rinsing with phosphate‐buffered saline (PBS), antigen retrieval was performed by heating the sections at an appropriate temperature for a prescribed duration in the heat retrieval solution Diva Decloaker (Biocare Medical) using a decloaking chamber (model DC2002, Biocare Medical). After blocking non‐specific binding with 10% goat serum for 15 min at room temperature, the sections were incubated overnight at 4°C with primary antibodies. The specificities of the primary antibodies used in this study were as follows: AQP4 (rabbit anti‐AQP4, 1:500; Santa Cruz Biotechnology), GFAP (rabbit anti‐GFAP, 1:1000; Proteintech), CD68 (mouse anti‐CD68, 1:50; DAKO), C9neo (mouse anti‐C9neo, 1:5000; Hycult Biotech) and ALDH1A2 (rabbit anti‐ALDH1A2, 1:2000; LSBio). The sections were then washed with PBS and incubated in 30% methanol/PBS containing 1% H_2_O_2_ for 20 min to block endogenous peroxidase activity, followed by three washes in PBS. Horseradish peroxidase‐conjugated secondary antibodies (Histofine Simple Stain Kit, Nichirei) were applied and incubated for 40 min at room temperature. Immunoreactivity was visualized using diaminobenzidine hydrochloride (DAB), and the sections were counterstained with hematoxylin. After staining, the sections were dehydrated through graded ethanol, cleared in xylene, and coverslipped using a mounting medium.

Rat tissue: Immediately after death by CO_2_ inhalation, the animals were perfused with 4% PFA and dissected to retrieve the CNS. The CNS was further fixed in 4% PFA for an additional 18–24 h, embedded in paraffin, and stored at room temperature (RT) until further use.

Both new and archival tissue blocks were used. All immunohistochemical procedures were performed as described [8], after antigen retrieval by steaming deparaffinized and rehydrated tissue sections for 1 h in 1 mM EDTA in 10 mM Tris buffer (pH 8.5), 10 mM citrate buffer (pH 6.0) or by incubating them for 15 min in 0.03% protease‐type XXIV, subsequently indicated by E, C, or P, respectively. The following primary antibodies were used for analysis: polyclonal rabbit anti‐rat aquaporin‐4 (AQP4; 1:250; E; Sigma‐Aldrich, Vienna, Austria), rabbit anti‐rat C9neo (1:2000; P [10]), polyclonal rabbit anti‐cow glial fibrillary acidic protein (GFAP; cross‐reactive with rat; 1:3000; E; Dako, Glostrup, Denmark), monoclonal mouse anti‐rat ED1 (1:10,000; E; Thermo Scientific, Vienna, Austria), rabbit anti‐ionized calcium‐binding adapter molecule 1 (Iba1; 1:3000; E; Wako, Neuss, Germany), rabbit anti‐Fc epsilon receptor 1 gamma (Fcer1γ; 1:1000; C; LifeSpan BioSciences, Newark, NJ), polyclonal goat anti‐human interleukin‐1 beta (IL‐1β, cross‐reactive with rat IL‐1β; 1:250; E; Santa Cruz Biotechnology, Dallas, TX), and rabbit anti‐aldehyde dehydrogenase 1 family member A2 (Aldh1a2; 1:1500; E; LifeSpan BioSciences). Primary antibodies were applied overnight at 4°C, followed by incubation with the corresponding biotinylated secondary antibodies (biotinylated donkey anti‐rabbit [1:1000–1:2000, Jackson ImmunoResearch], biotinylated donkey anti‐mouse [1:500, Jackson ImmunoResearch, West Grove, PA], and biotinylated donkey anti‐sheep/goat [1:500, Jackson ImmunoResearch]). Subsequently, avidin‐peroxidase was added, and reactions were completed with 3‐Amino‐9‐ethylcarbazole (AEC) (for C9 neo) or diaminobenzidine‐tetra‐hydrochloride (DAB, Sigma, Vienna, Austria) containing 0.01% hydrogen peroxide (all other antibodies).

For double labelling, biotinylated donkey anti‐goat (1:250; Jackson ImmunoResearch) and alkaline phosphatase donkey anti‐rabbit (1:100; Jackson ImmunoResearch) were used as secondary antibodies, and the reactions were completed by exposure to avidin‐peroxidase (1:500; Jackson ImmunoResearch) and subsequent visualization with Fast blue B salt (for Iba1; Sigma) and DAB (for IL‐1β; Sigma).

Finally, the tissue sections were directly mounted in geltol (double labelling), counterstained with hematoxylin and mounted in geltol (C9 neo), or counterstained with hematoxylin, dehydrated and mounted in Eukitt© (Merck, Darmstadt, Germany) (all other sections).

Both human and rat tissue sections were then scanned with a slide scanner (NanoZoomer Digital Pathology, Hamamatsu Photonics) and analyzed using Hamamatsu NDP.View2 software.

### 2.5 Multiplex immunofluorescent labelling

Labelling for markers of interest was performed using the Akoya Fluorescent Multiplex kit based on the manufacturer's protocol: The tissue sections were dewaxed in xylene for 30 min, transferred to ethanol and incubated in 0.2% hydrogen peroxide for 30 min to block endogenous peroxidase activity. Then, the sections were rehydrated through a graded ethanol series and rinsed in distilled water. Sections were first incubated with the primary antibody against AQP4 (rabbit anti‐AQP4, 1:500; Sigma‐Aldrich, Vienna, Austria) and developed using a peroxidase‐conjugated secondary antibody (1:200) followed by a 15‐min incubation with Opal 570 fluorophore (1:300 diluted in 1× Plus Amplification buffer). After development, sections were fixed in 4% paraformaldehyde (PFA) for 20 min at room temperature. Heat‐induced epitope retrieval (HIER) was performed by steaming in AR9 buffer for 1 h, followed by a 10‐min blocking step with Opal Antibody Diluent/Block solution. Between all steps, sections were washed with Tris‐buffered saline containing 0.1% Tween‐20 (TBST). Following the first staining cycle, a second primary antibody (rabbit anti‐rat 5‐Lipoxygenase (5‐Lo), 1:5000, Cell Signaling Technology, Danvers, MA) was applied, followed by incubation with the appropriate peroxidase‐conjugated secondary antibody (1:200) and Opal 690 fluorophore (1:300 in 1× Plus Amplification buffer). Then, sections were fixed in 4% PFA for 10 min at RT, and antigen retrieval was performed by steaming in AR6 buffer for 30 min, followed by a 10‐min blocking step with Opal Antibody Diluent/Block solution. The ED1 antibodies (mouse, 1:20,000; Thermo Scientific, Vienna, Austria) were applied and incubated overnight at 4°C. For each primary antibody, a corresponding secondary peroxidase‐conjugated antibody was applied. Opal fluorophores were used for signal detection, diluted in 1× Plus Amplification buffer, and applied to the sections for 15 min (Opal 620 for ED1).

An additional multiplex immunofluorescence staining was performed using the same Akoya Opal workflow described above, with modifications in the primary antibody combinations and fluorophore assignments. Sections were sequentially stained for vimentin, Aldh1a2, and GFAP. Vimentin was detected using a mouse anti‐vimentin antibody (1:5000; Dako, Glostrup, Denmark) and visualized with Opal 570. Aldh1a2 was detected using a rabbit anti‐Aldh1a2 antibody (1:10,000; LifeSpan Biosciences) and visualized with Opal 520. GFAP was detected using a rabbit anti‐GFAP antibody (1:15,000; Dako, Glostrup, Denmark) and visualized with Opal 690.

The nuclei were stained with 4′,6‐diamidino‐2‐phenylindole (DAPI). All Opal reagents and retrieval buffers were from Akoya Biosciences (Marlborough, MA), and secondary antibodies were from Jackson ImmunoResearch (West Grove, PA). The sections were mounted in geltol and scanned using the Vectra Polaris Automated Quantitative Pathology Imaging System (PerkinElmer).

### 2.6 Analysis of the stained tissue sections

Human tissue: Each lesion was classified into four groups according to the previously described astrocytopathy‐based staging system [5]. Lesions in the astrocyte lysis stage were defined as acute lesions, those in the progenitor recruitment stage as subacute lesions, and those in the protoplasmic gliosis stage and fibrous gliosis stage were collectively defined as chronic lesions. To evaluate parenchymal infiltration of microglia/macrophages, CD68‐immunostained sections were analyzed. Three circular regions of interest (ROIs), each measuring 0.025 mm^2^, were randomly selected within each lesion. The number of nuclei of CD68‐positive cells within each ROI was counted and expressed as the number of CD68‐positive cells per square millimeter.

Rat tissue: The staining of the sections was analyzed using the open‐source software Qupath‐0.5.1 [11]. For conventional immunohistochemistry, the area postrema and perivascular lesions were annotated, and reactivity to ED1, Iba1, or Fcer1γ was evaluated through the pixel classifier to quantify staining density for statistical analysis. The pixel classifiers were trained on DAB‐positive areas.

To quantify Aldh1a2 protein expression, the mean DAB optical density after immunohistochemical staining of Aldh1a2 was measured as regional staining intensity. Regions of interest (ROIs) were manually selected based on anatomical landmarks. For the overview analysis, fixed‐size ROIs of 0.041 mm^2^ were selected from different non‐AP CNS regions present on the same section, including medulla, pons, cerebellum, cerebral brain, and spinal cord. Because of the small size of the AP, the entire AP region was measured as one ROI. In addition, medulla sections at the level of the AP from control animals were used to show Aldh1a2 staining intensity between the AP and the surrounding medulla. The entire AP was measured as one ROI, while two ROIs of 0.041 mm^2^ were selected from the surrounding medulla. For each ROI, mean DAB optical density was extracted using QuPath intensity features, following the procedures described in the QuPath online user manual.

Fluorescently labeled cells were quantified semi‐automatically with QuPath, following the procedures described in the online user manual. This software provides a tool for machine learning‐based cell quantification. For every slide, channel separation was performed based on DAPI staining. The object classifier was subsequently trained and manually validated. After selecting a region of interest, the respective classifiers were applied to the data.

### 2.7 Statistics for IHC experiments

Statistical analysis was performed with nonparametric tests. For the comparison of multiple groups with controls, the Kruskal–Wallis test was used, followed by Bonferroni correction. A *p*‐value smaller than or equal to 0.05 was considered statistically significant. Statistical analyses and graphical visualizations were performed using SPSS 29.0.

### 2.8 Spatial transcriptomics

#### 2.8.1 Tissue preparation

Immediately after death, the animals were dissected, their upper cervical cord was frozen on dry ice for RNA preparation (quality control, see below), and the medulla was snap frozen in liquid nitrogen, using Tissue‐Tek (O.C.T.TM Compound; Sakuratek Finetek Europe B.V., Alphen aan den Rijn, NL) as embedding medium. The frozen material was stored at −80°C until further use.

#### 2.8.2 Quality control of mRNA

mRNA was isolated from the cervical cords using TRIzol (Thermo Fisher Scientific, Vienna, Austria) and the RNeasy mini Kit (Qiagen, Hilden, Germany) according to the instructions of the manufacturers. mRNA quality was then determined using the 2100 Bioanalyzer Instrument (Agilent Technologies, Vienna, Austria). In all cases, the RNA integrity number (RIN) was ≥9, indicating that our tissue preparations had intact mRNA and the material was suitable for gene expression analysis by spatial transcriptomics.

#### 2.8.3 Important information about methodology

Spatial transcriptomics was performed as an exploratory study, with *n* = 1 tissue section/experimental time point. Essentially, for this technique, a tissue section is placed on the capture area of a Visium spatial gene expression slide (10× Genomics B.V., LeCarrefour, Dellaertweg 9D, 2316 WC Leiden, the Netherlands). This capture area contains 5000 bar‐coded spots (i.e., 5000 spots with a unique “address tag”), with millions of capture probes per spot. When the tissue on top of these dots is permeabilized, the released RNA is immediately captured right underneath the site of release and translated to bar‐coded cDNA for sequencing reactions. At the end of this procedure, the bar‐codes (“the address tags”) allow for tracing the expressed genes back to the sites of RNA release, that is, to specific sites of the tissue section (https://www.10xgenomics.com/products/spatial-gene-expression). Identifications of these sites within the tissue is facilitated by HE staining of the tissue section used for spatial transcriptomics and by conventional immunohistochemical stainings of adjacent tissue sections.

In detail, the following experiments were performed at the Genomics and the Imaging Core Facilities of the Medical University of Vienna.

#### 2.8.4 Tissue optimization

This experiment was performed to determine the optimal time for permeabilization of tissue sections and liberation of RNA for spatial transcriptomics. The protocol on the 10× Genomics webpage (CG000238_VisiumSpatialTissueOptimizationUserGuide_Rev_A.pdf) was used. Briefly, 10 μm thick cryosections of control rat brain were used to test the efficiency of 3, 6, 12, 18, 24, and 30 min of tissue permeabilization. Imaging the fluorescence footprint testing of these six different timepoints and visual inspection of the image on an IX83 Live Imaging Microscope (Olympus) suggested 16 min as optimal permeabilization time, which was then used on the spatial gene expression slides.

#### 2.8.5 Visium spatial gene expression analysis

Visium spatial transcriptomics (ST) experiments followed the current protocols published on the 10× Genomics webpages (see below). Briefly, sections from rat medulla at the level of the area postrema were used, one each from the following treatments: Day 0 (neg. control), mAQP4‐ab present for 1 and 2 days. Sections were cut in a pre‐cooled cryostat at 10 μm thickness onto a Visium Spatial Gene Expression Slide (PN:2000233/SN:V12U07‐300 from 10× Genomics B.V., LeCarrefour, Dellaertweg 9D, 2316 WC Leiden, the Netherlands) with four 6.5 mm × 6.5 mm capture areas each containing ~5000 oligo‐barcoded spots. Each spot covered a 55‐μm area with a 100‐μm center‐to‐center distance. Slides then underwent fixation and HE staining using the protocol CG000160_DemonstratedProtocol_MethanolFixationandHEStaining_RevC.pdf on the 10× Genomics webpage. The stained sections were immediately imaged on an IX83 Live Imaging Microscope (Olympus) at 10× magnification. The high‐resolution images were used as input for Space Ranger without further adjustment. Space Ranger is a set of analysis pipelines that process 10× Genomics Visium data with microscope images (https://www.10xgenomics.com/support/software/space‐ranger/latest). All subsequent reactions were carried out using the protocol CG000239_VisiumSpatialGeneExpression_UserGuide_RevF.pdf on the 10× Genomics webpage. The resulting DNA libraries were then sequenced on the NextSeq2000 VH00748 platform and 230512_VH00748_35_AAAYJ7THV Flowcells according to the Visium Spatial Gene Expression Reagent Kits User Guide (10× Genomics protocol), Version F. The 10× Genomics protocol recommended a sequencing depth/spot of at least 50,000 read pairs per tissue covered spot on the capture area. Our tissue samples covered 50% (for medulla of control animal [=animal A], in which mAQP4‐abs were present for 0 days), and 55% (for medullae of animals in which mAQP4‐abs were present for 1 day [giving rise to patchy AQP4 loss in the area postrema, animal B] and 2 days [giving rise to complete AQP4 loss in the area postrema, animal C]) of the capture area. The sequencing depth/sample was than calculated according the following formula, here given as example calculation for 55% coverage: 0.55 × 5000 total spots × 50,000 read pairs/spot = 138 million total read pairs for that sample. The mean reads/spot achieved was 72,453 (medulla of animal A), 55,948 (medulla of animal B), and 58,172 (medulla of animal C), which corresponded well to the amount of reads per spot recommended in the 10× Genomics protocol. In Space Ranger 2.0.1, the raw sequencing data were demultiplexed and processed using the Space Ranger mkfastq command to generate read sequences which were mapped against the rat reference genome version rnor.6.0, restricting the genes to be included to biotype “protein_coding.” This generated a dataset with a median of 2775 (medulla of animal A), 2702 (medulla of animal B), and 3122 (medulla of animal C) genes detected per tissue covered spot. Here, two types of quality control were included. First, a search for transcripts in spots outside the tissue which turned out negative, indicating that there was no leakage of transcripts in the tissue permeabilization and mRNA capture processes. Second, an analysis of the reads quality using FASTQC, which revealed a mean quality score of 32, indicating a very good quality of the reads. Using the Space Ranger count command for analysis of “spots covered by tissue,” gene expression data per gene and spot were obtained and displayed against the images of the HE‐stained tissue sections.

#### 2.8.6 Data analysis and statistics

Space Ranger output data (cloupe.cloupe file) were analyzed in Loupe Browser 6.5.0 and Loupe Browser 7.0 (https://www.10xgenomics.com/support/software/loupe-browser/latest) for cluster definition and data export.

Visualization of the location of specific gene expression was carried out in Loupe Browser by selecting “gene/features expression.” Defining areas of interest on the tissue section was carried out manually using the selection tools. For each medullary section, differentially upregulated genes within the defined area were identified in Loupe Browser by selecting the “categories” function and “globally distinguishing” from the “significant feature comparison” function. Using the table option “hide genes with low average count,” we set very strict cut‐off criteria and only considered features with an average occurrence greater than 1 count per cell across the entire dataset, which filtered out relatively rare features which may still yield small *p*‐values in the differential expression calculation between clusters. Using these settings, we were able to identify genes in the area postrema and in the inflamed parenchymal perivascular area, which showed upregulated expression compared to the remaining medulla of the same tissue section.

Statistical analysis was performed by Space Ranger and essentially compared gene expression of a selected area (e.g., the area postrema) to the rest of tissue (e.g., the medulla) on the same section. It included the calculation of Moran's *I* values for each gene. Moran's *I* is a correlation coefficient for spatial autocorrelation and measures how one object is similar to others surrounding it. Moran's *I* values range from −1 to 1 (the closer to 1 the values are, the better enriched the gene products are at a given site). The statistical significance of Moran's *I* values was determined with a simple hypothesis test, calculating a *z*‐score and its associated *p*‐value. Space Ranger provided us with the Moran's *I* values, the *p*‐values, and the *p*‐values corrected using the Benjamini–Hochberg method for multiple comparisons.

### 2.9 Functional annotation clustering

Functional annotation clustering of differentially expressed transcripts was made with the Database for Annotation, Visualization and Integrated Discovery (DAVID; https://david.ncifcrf.gov/, version December, 2024 [12, 13]), using the official_gene_symbol of Rattus norvegicus of genes with statistical significance of *p* < 0.05 (adjusted using the Benjamini–Hochberg correction for multiple tests). Doing this, LOC genes were excluded from the input list unless they could be translated to gene symbols. For our studies, we used the DAVID annotation chart assembled for the Gene Ontology Terminus “biological processes” (GOTERM‐BP). From the top 40 functional clusters, we selected those known to affect (i) neurons and glia and (ii) immune cells and microglia. The EASE Score (=alternative name of Fisher Exact Statistics in DAVID system, a one‐tail Fisher Exact probability value used for gene‐enrichment analysis; https://david.ncifcrf.gov/helps/functional_annotation.html) was used to determine the enrichment of genes in the annotation terms, with *p*‐values equal or smaller than 0.05 indicating strong enrichment.

The Human Protein Atlas (https://www.proteinatlas.org/) was used to obtain initial information about gene names, function, and possible expression of gene products in cells and tissues [14].

### 2.10 snRNAseq analysis

For single‐nuclei RNA sequencing (snRNAseq) analysis, published raw count tables from the rat area postrema (GSE167981, GSE167991) were retrieved [15]. The count tables were merged and processed with the Seurat pipeline [16, 17]. After dimensional reduction with principal component analysis (PCA) and Uniform Manifold Approximation and Projection (UMAP) and clustering with the Leiden algorithm [18], the expression levels of 14 different molecules (Cx3cr1, Cldn5, Vim, Notch2, Slc1a3, Cfap52, Cldn10, Pcdh15, Mag, Snca, Scl5a7, Slc17a6, Gad1, and Snap26) were used to annotate clusters of distinct cell types within the CNS as described [15] (Figure S1, Supporting Information). These clusters were searched for specific gene expression patterns (Figure 3). The dataset was then saved as an hdf5 file for further use in a python pipeline. The detailed Jupyter notebook can be found here: https://github.com/TBouderlique/area_postrema.

### 2.11 Spatial RNAseq analysis

Briefly, the reads produced by spatial transcriptomics as described above were processed with Space Ranger (v1.3.1, 10× Genomics). Raw counts were then processed with the Scanpy [19] and Squidpy [20] pipelines. Using the snRNAseq as a reference, spatial cell type scores were calculated with Tangram [21]. The detailed notebooks used to analyze the data are available here: https://github.com/TBouderlique/area_postrema.

## 3 RESULTS

### 3.1 The clinical course of area postrema syndrome in NMOSD patients

We analyzed 39 NMOSD patients with a total number of 71 area postrema syndrome (APS) attacks defined by hiccups (49/71), nausea (40/71), vomiting (20/71), and coughing (3/71). Clinical information at the end of the acute phase (30 days after the onset of APS) was available for 58 attacks. 45/58 had received high‐dose intravenous methylprednisolone therapy (1000 mg/day for 3 days), and 7/58 were treated with oral prednisolone. Including cases with no immunotherapy, APS eventually disappears in 95% of total cases (5% residual APS; Table 1).

**FIGURE 1:**
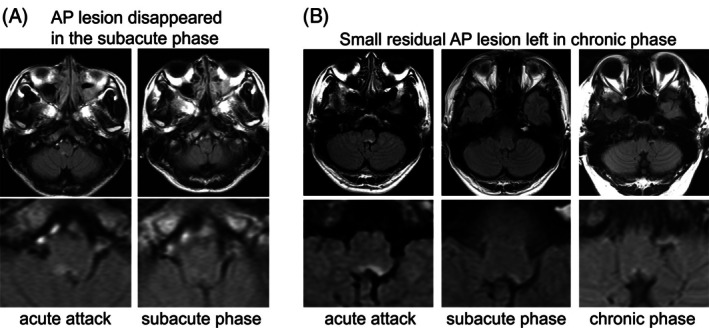
Temporal changes in MRI findings of area postrema lesions in patients with NMOSD. (A) A FLAIR hyperintensity lesion in the area postrema during an acute attack (left), disappeared after treatment (subacute phase, right). (B) A FLAIR hyperintensity lesion in the area postrema decreasing in size over time. It is still visible in the chronic phase of the disease, but is not accompanied by tissue atrophy.

Acute area postrema lesions were confirmed by MRI in 34/52 attacks, and the fate of these lesions could be followed over time in 15 patients by further imaging analysis. In 3/15 patients, the lesions disappeared in the subacute phase (i.e., within 6 months after attack; Figure 1A), in 6/15 patients, they were still present in the subacute phase but disappeared in the chronic phase (i.e., more than 6 months after attack), and in 5/15 patients, MRI was not performed in the subacute phase, but lesions were gone in the chronic phase. Only 1/15 patients had a residual area postrema lesion left in the chronic phase, but with much smaller size than seen during acute attack (Figure 1B). These clinical and imaging features of APS were clearly different from those of myelitis in NMOSD, where approximately three‐quarters of cases experience residual symptoms [22] and where complete resolution of spinal cord lesions on MRI is extremely rare [23].

**FIGURE 2:**
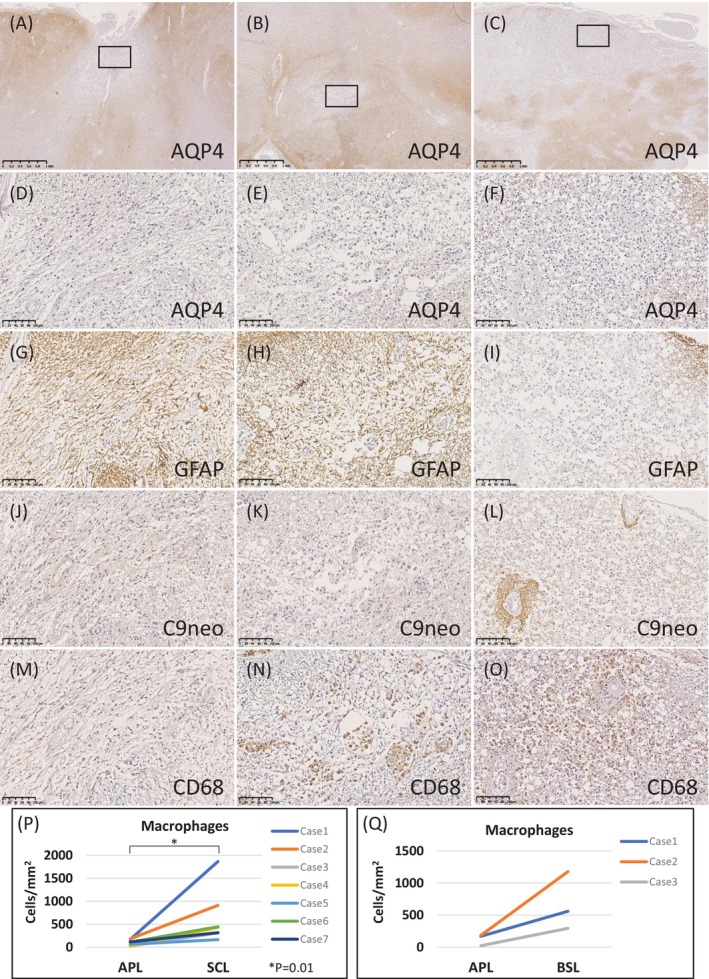
Histological comparison of area postrema, brainstem, and spinal cord lesions in human tissues. All immunoreactivities are shown in brown. Sections were counterstained with hematoxylin to visualize nuclei in blue. All tissue sections shown are derived from Case 1. (A) Area postrema lesion. (D) Higher magnification of the boxed area in panel (A); (G, J, M) the corresponding area shown with different immunostainings. AQP4 immunoreactivity was lost (A, D), whereas astrocytic fibers were still present (G). No C9neo deposition was observed (J). Only a few CD68‐positive macrophages/microglia were present within the lesion (M). (B) Typical chronic lesion in the brain stem. (E) Higher magnification of the boxed area in panel (B); (H, K, N) the corresponding area shown with different immunostainings. Note the loss of AQP4 immunoreactivity (B, E) and the presence of GFAP immunoreactivity (H), indicating astrocyte regeneration. C9neo deposition was not observed (K). CD68‐positive macrophages were present within the parenchyma (N). (C) Typical acute lesion in the spinal cord. (F) Higher magnification of the boxed area in panel (C); (I,L, O) the corresponding area shown with different immunostainings. AQP4 (F) and GFAP (I) immunoreactivity was lost. C9neo deposition was observed in a rosette‐like pattern around blood vessels (L) and CD68‐positive macrophages/microglia were present (O). Scale bars = 100 μm. (P) Comparison between paired area postrema lesions (APLs) and spinal cord lesions (SCLs). Macrophage density was consistently lower in APLs than in corresponding SCLs. (Q) Comparison between paired APLs and brain stem lesions (BSLs). Macrophage density also tended to be lower in APLs than in corresponding BSLs. Each line represents an individual case.

We next conducted a histopathological analysis of 18 autopsied NMOSD cases. Among them, medulla oblongata tissue containing the area postrema was available for analysis in 10 cases, and AQP4‐loss lesions in the area postrema (APL) (Figure 2A,D) were identified in seven of these cases (Table 2). In three of the seven APL, partial loss of GFAP‐positive astrocytes was observed; however, complete astrocyte destruction was not identified in any lesion (Figure 2G). Tissue damage was generally mild, and no C9neo deposition was observed in any APL with AQP4 loss (Figure 2J). In addition, infiltration of CD68‐positive macrophages/microglia was sparse (Figure 2M), with a mean density of 96.2 ± 66.7/mm^2^. We further analyzed brainstem lesions outside the area postrema (BSL) in 11 cases and spinal cord lesions (SCL) in all 18 cases. In contrast to APL, pathological features of BSL and SCL were largely similar. In all seven acute‐to‐subacute lesions, severe necrotic tissue damage associated with extensive AQP4 loss (Figure 2C,F) and GFAP loss (Figure 2I) was observed, indicating marked astrocyte destruction. Characteristic perivascular C9neo deposition was identified in three acute‐stage lesions (Figure 2L), accompanied by abundant CD68‐positive macrophages/microglia (Figure 2O). In chronic lesions, AQP4 immunoreactivity remained lost (Figure 2B,E) despite regeneration of astrocytes within the lesions (Figure 2H). Although C9neo deposition was no longer detectable in chronic lesions (Figure 2K), relatively abundant CD68‐positive macrophages/microglia were still present (Figure 2N).

Overall, these findings suggest that the human area postrema shows relative resistance to AQP4 antibody‐mediated tissue injury compared with other CNS regions affected in NMOSD.

### 3.2 Core NMOSD features are reproduced in the AQP4 abs‐driven experimental rat model

**FIGURE 3:**
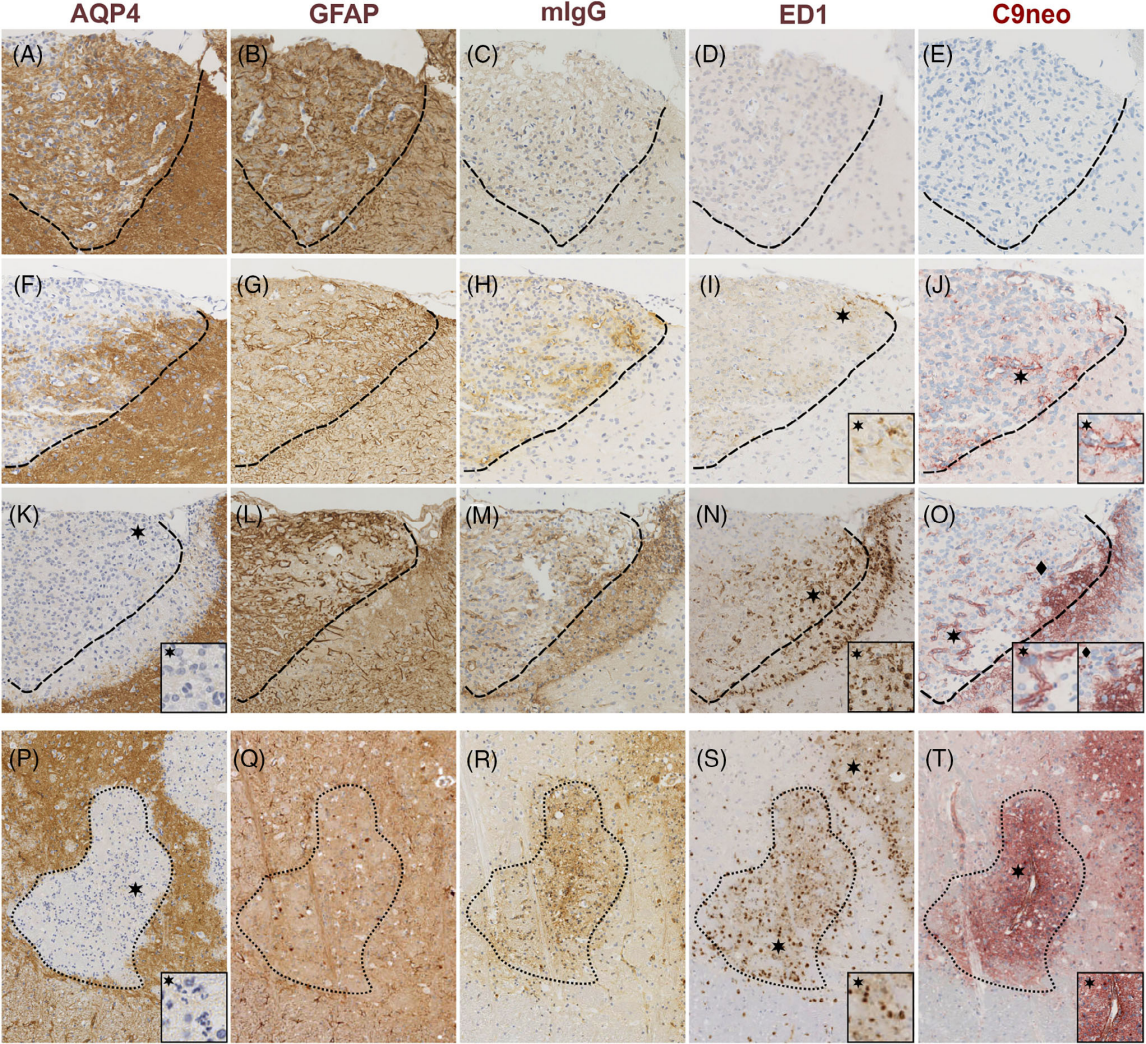
Characterization of mAQP4‐abs induced changes in Lewis rats. Immunohistochemical analysis with antibodies against aquaporin‐4 (AQP4), glial fibrillary acidic protein (GFAP), and murine IgG (mIgG, detecting the murine E5415A antibody), the ED1 antibody recognizing CD68+ activated microglial cells/macrophages, and antibodies against C9neo were made. Please note that all positive reaction products of immunohistochemical stainings are shown in brown (AQP4, GFAP, mIgG, ED1) or red (C9neo), and that the sections have been counterstained with hematoxylin to show nuclei in blue. (A–E) intact area postrema; (F–J) area postrema with patchy loss of AQP4 reactivity; (K–O) area postrema with complete loss of AQP4 reactivity. Black broken line indicate outline of the area postrema; (P–T) perivascular lesion in the medulla distant from the area postrema. Black dotted line indicates the lesion outline. *Areas shown in higher magnification in the lower right corner of the figures to demonstrate the absence (K) or presence (P) of neutrophils with their characteristic lobulated nuclei; and to better see ED1+ cells (I, N, S) and C9neo deposition (J, O, T).

The intact area postrema of unmanipulated Lewis rats is characterized by AQP4 and GFAP expression (Figure 3A,B, respectively) and by the absence of immunoglobulin deposition (Figure 3C), absence of microglia/macrophage activation (as evidenced by a lack of ED1 reactivity; Figure 3D), and the absence of membrane attack complexes (as evidenced by lack of C9neo reactivity; Figure 3E).

The first type of area postrema pathology was seen in 3/4 animals systemically challenged with AQP4‐abs for 24 h and in 2/5 animals challenged for 48 h, and was characterized by a patchy loss of AQP4 reactivity in the area postrema (Figure 3F), in the absence of GFAP loss (Figure 3G), indicative for AQP4 internalization upon antibody binding and survival of the affected cells.

Patchy deposition of immunoglobulin was noted (Figure 3H), in the absence of extensive microglial activation or of macrophage recruitment (as evidenced by ED1 staining; Figure 3I). At the border of the area postrema, C9neo reactivity was observed at the perivascular glia limitans (Figure 3J). Within the area postrema parenchyma, either no or locally confined and weakly punctuated complement deposition adjacent to the C9neo‐reactive perivascular glial limitans was noticed (Figure 3J).

The second type of area postrema pathology was seen in 1/4 the animals seropositive for mAQP4‐abs for 24 h, 3/5 animals seropositive for 48 h, and 5/5 animals seropositive for 96 h, and was characterized by a complete loss of AQP4 reactivity in the area postrema (Figure 3K). At the border of the area postrema, close to the funiculus separans, AQP4 (Figure 3K) and GFAP (Figure 3L) reactivities were lost and immunoglobulin deposition (Figure 3M), large numbers of ED1^+^ ameboid microglial cells/macrophages (Figure 3N), and C9neo deposits (Figure 3O) were seen, suggesting local tissue damage because of ADCC and CDC. This type of pathology seemed to spread from the border area inwards, and the exact mechanisms for this finding are still subject to further investigations. In the center of the area postrema, AQP4 reactivity was lost (Figure 3K), but GFAP reactivity (Figure 3L) was present. This pattern suggests internalization of AQP4 upon mAQP4‐abs binding and survival of the affected cells. Immunoglobulin deposition at the perivascular glia limitans was seen (Figure 3M), and ED1+ microglial cells/macrophages (Figure 3N) were present. Complement C9neo reactivity (Figure 3O) was either completely absent or weakly punctuated.

Medullary lesions (Figure 3) and spinal cord lesions (Figure S1) were characterized by loss of AQP4 (Figure 3P) and GFAP reactivity (Figure 3Q), by leakage of mAQP4‐abs into the parenchyma (as evidenced by high reactivity of murine immunoglobulin distributed throughout the lesions; Figure 3R) and by large numbers of neutrophils (Figure 3P, inset) and many activated amoeboid ED1+ microglia/macrophages (Figure 3S). In line with the profound deposition of C9neo in the medullary lesions (Figure 3T), the pathological picture at this site reflects death of astrocytes by ADCC and CDC [8].

### 3.3 The molecular and immunological landscape of the intact area postrema

Both in NMOSD patients and in animals with experimental NMOSD, the area postrema is more resilient to mAQP4‐abs mediated damage than other sites of the CNS. Therefore, we used CNS tissue of Lewis rats to search for molecular cues contributing to the resilience of the area postrema by spatial transcriptomics and subsequent functional annotation clustering with DAVID.

**FIGURE 4:**
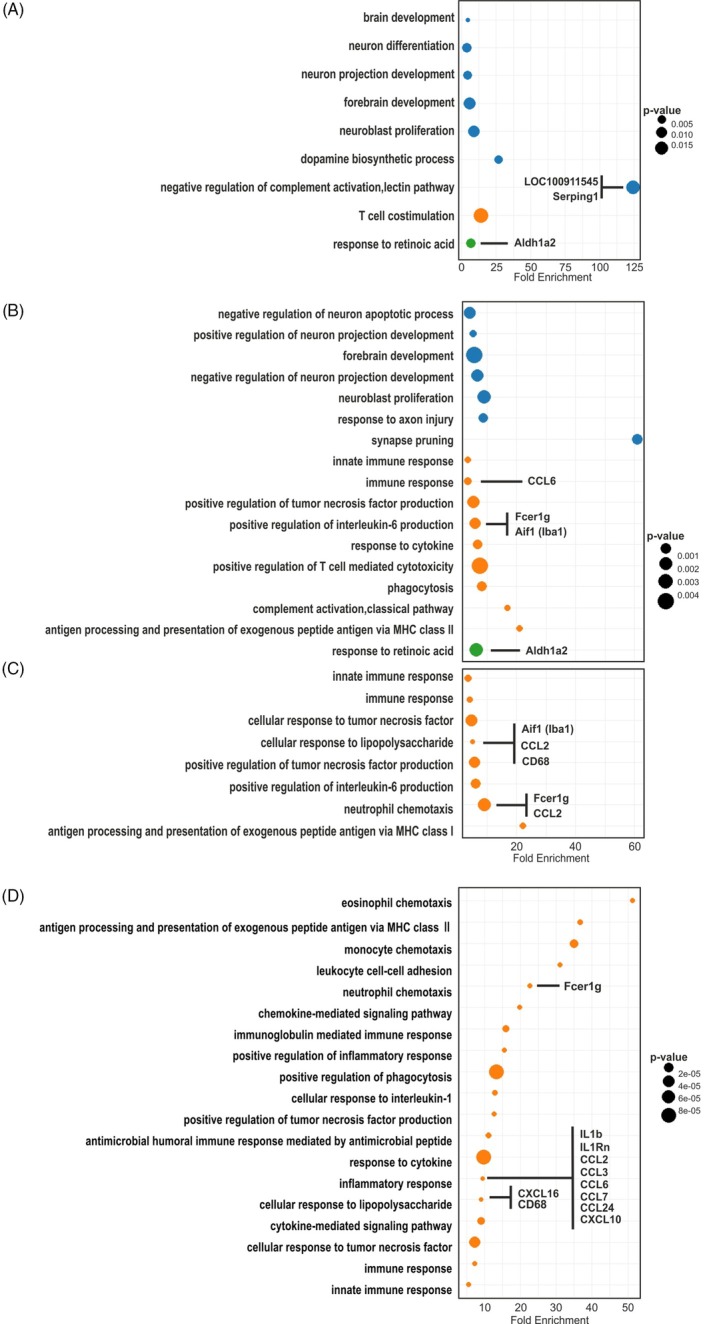
GO analysis of genes upregulated in the intact area postrema of a healthy, untreated Lewis rat (A), in the area postrema with patchy loss of AQP4 reactivity (shown here are data from a Lewis rat challenged with mAQP4‐abs for 24 h (B)), in the area postrema with complete loss of AQP4 reactivity (shown here are data from a Lewis rat challenged with mAQP4‐abs for 48 h (C)), and in medullary lesions with AQP4 loss (from a Lewis rat challenged with mAQP4‐abs for 48 h (D)).

**FIGURE 5:**
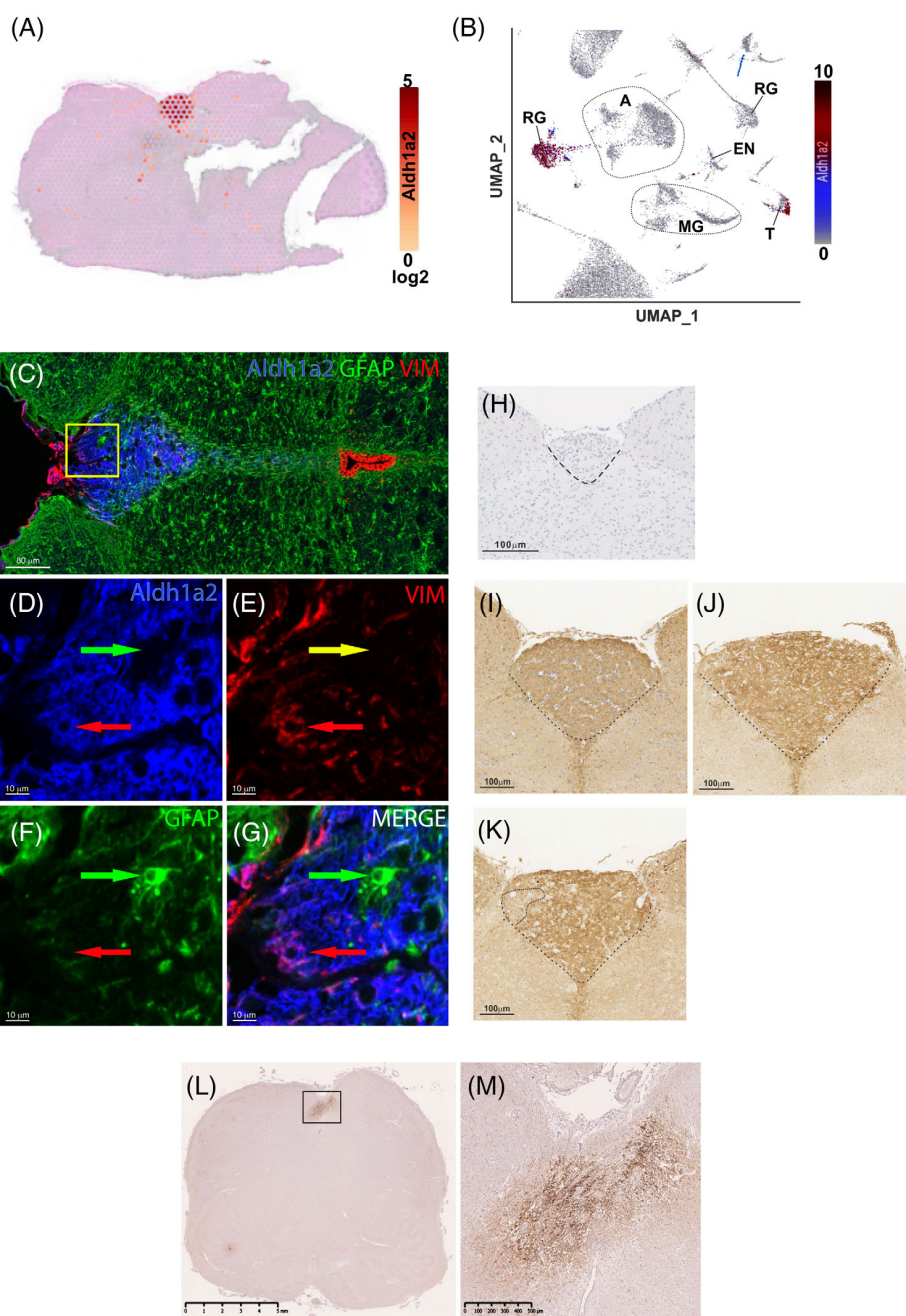
Expression of aldehyde dehydrogenase 1 family member A2 (Aldh1a2) in the area postrema. (A) Loupe Browser image of the medulla of a healthy, untreated Lewis rat, sectioned at the level of the area postrema. The red dots in this tissue section are located on top of the area postrema, and each of the dots represents a bar‐coded spot in which Aldh1a2 transcripts were upregulated. The color scale shows the log2 expression levels of the Aldh1a2 transcripts. (B) Uniform Manifold Approximation and Projection embedding of snRNA‐seq data from the area postrema and the nucleus tractus solitarius (*n* = 8 rats, GSE167981, GSE167991 [15]) and cell type assignment using gene‐based clusters (see Figure S1). RG = radial glial cells, A = astrocytes, EP = ependymal cells, MG = microglial cells, T = tanycytes, EN = endothelial cells. High amounts of Aldh1a2 transcripts (red) were found in radial glial cells and tanycytes. (C–G) Multiplex staining of the medulla from a 5‐month‐old Lewis rat. The medulla section was made at the level of the area postrema. The merge of all stainings is shown in (C). The yellow square defines an area for which stainings for Aldh1a2 (blue, D), Vim (red, E) GFAP (green, F), and a merge of these 3 stainings (G) is shown in higher magnification. The green arrows point to a GFAP^High^ Aldh1a2^−^ cell, the red arrows point to a Vim^+^ GFAP^−^ Aldh1a2^High^ tanycyte. (H–K) Immunohistochemical analysis of the area postrema (dashed outline). (H) Intact area postrema of an animal which had not been treated with E5415A. For immunohistochemical control, the anti‐ALdh1a2 antibody was omitted during staining (data representative for 2 animals studied). (I–K) Immunhistochemical detection of Aldh1a2 protein in the rat area postrema. (I) Intact area postrema of an animal which had not been treated with E5415A (data representative for 17 animals studied). (J) Area postrema with patchy loss of AQP4 reactivity (data representative for 5 animals). (K) Area postrema with complete loss of AQP4 reactivity, and a small area adjacent to the area postrema border with astrocyte loss (dotted outline line) (data representative for 9 animals). (L, M) Medulla of an NMOSD patient (case 1), stained for Aldh1a2 protein. Please note that Aldh1a2 is very strongly expressed in the area postrema (F, boxed area shown in g in higher magnification).

Gene products upregulated in the intact area postrema were involved in “brain development” or “forebrain development,” neurotransmitter “(Dopamine) biosynthetic process,” “neuron projection development,” “neuron differentiation,” and “neuroblast proliferation” (Figure 4 and Table S1A). These findings are in line with the recent discovery of the area postrema as a neurogenic stem cell niche in adult animals mammals [24]. We also observed an annotation of the gene cluster “response to retinoic acid” (Table S1A) which contained aldehyde dehydrogenase 1 family member A2 (Aldh1a2), a key molecule for the synthesis of retinoic acid (RA) from retinaldehyde [25]. Aldh1a2 transcripts showed a log_2_ fold change of 5 in the area postrema compared to the medulla (Figure 5A), which means that the expression of this molecule is 32 times (2^5^) higher in the area postrema than in the medulla. We further compared Aldh1a2 protein expression in the area postrema to Aldh1a2 protein levels elsewhere in the CNS (Figure S3) by determining the optical density of the DAB product after immunohistochemical analysis of different CNS areas on the same tissue slide. Despite a relatively high background reactivity of these sections, higher levels of Aldh1a2 proteins in the area postrema compared to medulla, other brain areas, and spinal cord (Figure S3) were seen.

We next set out to determine the cell types producing Aldh1a2 in the area postrema and compared our newly generated spatial transcriptomics data with a previously generated data set of single‐nuclei RNA sequencing results from the rat area postrema and nucleus tractus solitarius [15], which allowed us to search for Aldh1a2 transcripts in unambiguously defined cell populations of the area postrema. We found Aldh1a2 transcripts in Vim^+^ tanycytes and in Slc1a3^+^, Cldn10^−^, Vim^low^, and Notch^low^ radial glial cells (Figure S2 and Figure 5B). We also performed multiplex stainings and could further confirm cytoplasmatic Aldh1a2 protein expression in the rat area postrema, show its expression in some Vim + tanycytes, and exclude GFAP^high^ astrocytes as a major source of Aldh1a2 expression in the area postrema, compatible with the expression of this molecule in local radial glial cells (Figure 5C–G). High Aldh1a2 protein expression was seen both in the intact (Figure 5I) and in the AQP4‐abs challenged area postrema with patchy (Figure 5J) and complete AQP4 loss (Figure 5K).

In human control tissues, Aldh1a2 immunoreactivity was examined across multiple rostrocaudal levels of the medulla. In the upper and middle medulla, where the fourth ventricle remained widely open, only minimal scattered Aldh1a2 immunoreactivity was occasionally observed near the ventricular surface without forming distinct anatomical structures (Figure S4A–D). In contrast, clear Aldh1a2‐positive staining appeared in restricted dorsal regions of the lower medulla as the fourth ventricle narrowed toward the obex (Figure S4E–J). At levels corresponding anatomically to the area postrema near the obex, bilateral Aldh1a2‐positive regions became more prominent and converged toward the midline (Figure 5). In more caudal sections near the central canal transition, the Aldh1a2‐positive structure became smaller (Figure S4K,L). Although the staining intensity was relatively similar across different medullary levels, the size of the Aldh1a2‐positive structures varied rostrocaudally. Similar localization patterns were also observed in an ALS case (Figure S4M,N).

Finally, spatial transcriptomics also identified two upregulated genes in the cluster “negative regulation of complement activation, lectin pathway”: The first one was LOC100911545 (Tables S1A and S1B; according to information of the rat genome database (RGD) initially assigned as “alpha‐2‐macroglobulin‐like,” and replaced by the gene A2m on March 9, 2021; https://rgd.mcw.edu/rgdweb/report/gene/main.html?id=6492449). Unfortunately, however, the Ensembl ID numbers of LOC100911545 (ENSRNOG00000028896), A2m (ENSRNOG00000045772), and A2ml (ENSRNOG00000007247) probed for in spatial transcriptomics did not match; we did not find transcripts for A2m and A2ml in our samples, and the commercially available antibodies raised against A2m did not stain anything in our tissue sections. Therefore, we could not confirm this molecule on the protein level. The second transcript of the cluster “negative regulation of complement activation, lectin pathway” was Serping1 (Table S1A), and we could ascribe these molecules to radial glial cells/astrocytes (data not shown). However, we were also unable to confirm Serping1 on the protein level, possibly because of expression levels below the limit of detection by immunohistochemistry. Therefore, we could not further study the role of the cluster “negative regulation of complement activation, lectin pathway” for the resilience of the area postrema to mAQP4‐abs mediated damage.

Of note, we did not observe any cluster containing the complement regulators CD46, CD55, and CD59 (Table S1A). Gene products with significantly lower expression in the area postrema than in the medulla were essentially involved in “myelination”/“myelin formation” and encompassed myelin associated glycoprotein (MAG), myelin basic protein (MBP), and myelin associated oligodendrocyte basic protein (MOBP) (Table S1B).

### 3.4 The molecular and immunological landscape of the AQP4‐abs challenged area postrema

In the area postrema with patchy loss of AQP4 reactivity, gene transcripts involved in “neuroblast proliferation” and “forebrain development” were still detectable, while transcripts in the clusters “negative” and “positive regulation of neuron projection development,” “synapse pruning,” “response to axon injury” became upregulated (Figure 4B and Table S2A). These changes were not yet evident in immunohistochemical stainings using antibodies against amyloid precursor protein (APP; data not shown), possibly because of low numbers of affected neurons or because of their small size which made it impossible to clearly identify axonal spheroids indicative of neuronal dysfunction/damage.

The cluster of “response to axon injury” also included transcripts for colony stimulating factor 1 receptor (Csfr1), transmembrane immune signaling adaptor (Tyrobp), AXL receptor tyrosine kinase, and allograft inflammatory factor 1 (Aif1), also named ionized calcium‐binding adapter molecule 1 (Iba1), which all involved microglial cells/macrophages. Activation of these cells was further evidenced by the clusters “innate immune response,” “phagocytosis,” and “antigen processing and presentation of exogenous peptide antigen via MHC class II molecules.”

The cluster “immune response” contained the only chemokine transcripts detectable, that is, CCL6 (Table S2A). We also detected evidence for the “positive regulation of interleukin‐6 production” and for the “positive regulation of tumor necrosis factor production,” while transcripts for Il6, Tnf, and Il1‐β could not be detected. One important molecule shared by several of the identified clusters was Fc epsilon receptor I gamma (Fcer1γ) (Table S2A) which is not only part of the high affinity immunoglobulin E (IgE) receptor, but also a subunit of other Fc receptors found on cells of the innate immune system [26].

We also detected upregulated transcripts of the earliest steps of “complement activation, classical pathway” which included molecules encoding C1qa, C1qb, and C1qc involved in synapse pruning, but also revealed an upregulation of transcripts for C3, C1s, and Serping1.

Finally, we found the cluster “response to retinoic acid” with transcripts for Aldh1a2 (Table S2A). Aldh1a2 could also be confirmed at the protein level in the rat area postrema (Figure 5J).

Gene products with significantly lower expression in the area postrema with patchy loss of AQP4 reactivity compared to the medulla were essentially involved in myelination, neuronal development, differentiation, and function (Table S2B).

In the area postrema with complete loss of AQP4 reactivity, we also detected many upregulated gene products affecting immune cells and microglia, clustered within the complexes “cellular response to lipopolysaccharide,” “immune response” and “innate immune response,” and “antigen processing and presentation of exogenous peptide antigen via MHC class II” (Table S3A and Figure 4C).

We also found the clusters “positive regulation of interleukin‐6 production,” “positive regulation of tumor necrosis factor production,” “cellular response to tumor necrosis factor,” and “neutrophil chemotaxis.” We only detected upregulation of transcripts for Ccl2 (Table S3A), but not of those encoding Ccl6 or any other CC‐, CXC‐, or CX3C‐chemokines, suggesting only limited recruitment of immune cells from outside the area postrema.

8/top 40 functional clusters contained transcripts for Aldh1a2, the key molecule for retinoic acid synthesis (Table S3A), which could be further confirmed at the protein level (Figure 5K). We did not find any significantly downregulated genes in the area postrema with complete loss of AQP4 reactivity compared to the medulla (Table S3B).

A cardinal feature of all AQP4‐ab challenged area postrema in the Lewis rat was the expression of Aldh1a2 (Figure 5). We therefore also studied the area postrema of human NMOSD patients at different timepoints of disease in more detail. We examined APLs from seven NMOSD cases. In all lesions, ALDH1A2 immunoreactivity was preserved or even slightly increased compared with normal control tissues despite complete loss of AQP4 immunoreactivity. Cases 1–3 represented active disease, as acute to subacute lesions were identified in other CNS regions. In these cases, AQP4‐loss lesions extended slightly beyond the ALDH1A2‐positive areas (Figure S5A–H). In contrast, Cases 4–7 represented non‐active disease, as more than 1 year had passed because the last clinical relapse and only chronic lesions were identified outside the area postrema. In these cases, the areas of AQP4 loss largely corresponded to the ALDH1A2‐positive regions (Figure S5I–L). The intensity of ALDH1A2 immunoreactivity was generally similar among APLs in NMOSD. In MS cases, AQP4 immunoreactivity within the area postrema was preserved, and the pattern of ALDH1A2 immunoreactivity was largely comparable to that observed in normal control tissues (Figure S5M–P).

### 3.5 The molecular and immunological landscape of AQP4‐abs induced lesions in the medulla

Adjacent to the area postrema with complete loss of AQP4 reactivity, a deep parenchymal perivascular lesion in the medulla was large enough for characterization by spatial transcriptomic analysis and functional annotation clustering with DAVID. The top 40 functional clusters contained those with transcripts involved in “inflammatory and innate immune responses,” “positive regulation of inflammatory responses,” “immunoglobulin mediated immune responses,” “antimicrobial humoral immune responses mediated by antimicrobial peptide,” “positive regulation of phagocytosis,” “positive regulation of tumor necrosis factor production,” “chemotaxis of eosinophils, neutrophils, and monocytes,” and “antigen processing and presentation of exogenous peptide antigen via MHC class II” (Figure 4D and Table S4A). Most strikingly, and in marked contrast to what we saw in the area postrema of the same animal, the top 40 annotated gene clusters of this lesion contained many transcripts for chemokines, like the CC‐chemokines CCL2, CCL3, CCL6, CCL7, CCL9, CCL24, and the CXC‐chemokines CXCL10 and CXCL16. Moreover, the IL1‐β pathway was activated, as evidenced by upregulation of transcripts of IL1‐β (Figure 4D), and of the IL1 receptor antagonist (IL1rn), and by the “cellular response to interleukin‐1” ranking at position 18 of all annotated gene clusters (Table S4A). Additionally, “cellular responses to tumor necrosis factor” were seen (Table S4A).

None of the top 40 clusters contained Aldh1a2. We did not find evidence for a significant downregulation of genes in perivascular regions compared to the rest of the medulla (Table S4B).

### 3.6 Key differences in the activated microglia/macrophage and neutrophil cell populations between the AQP4‐abs challenged area postrema and medullary lesions

Because microglia/macrophages dominate the immune response in the mAQP4‐abs challenged area postrema and medullary lesions, we next characterized these cells in more detail.

**FIGURE 6:**
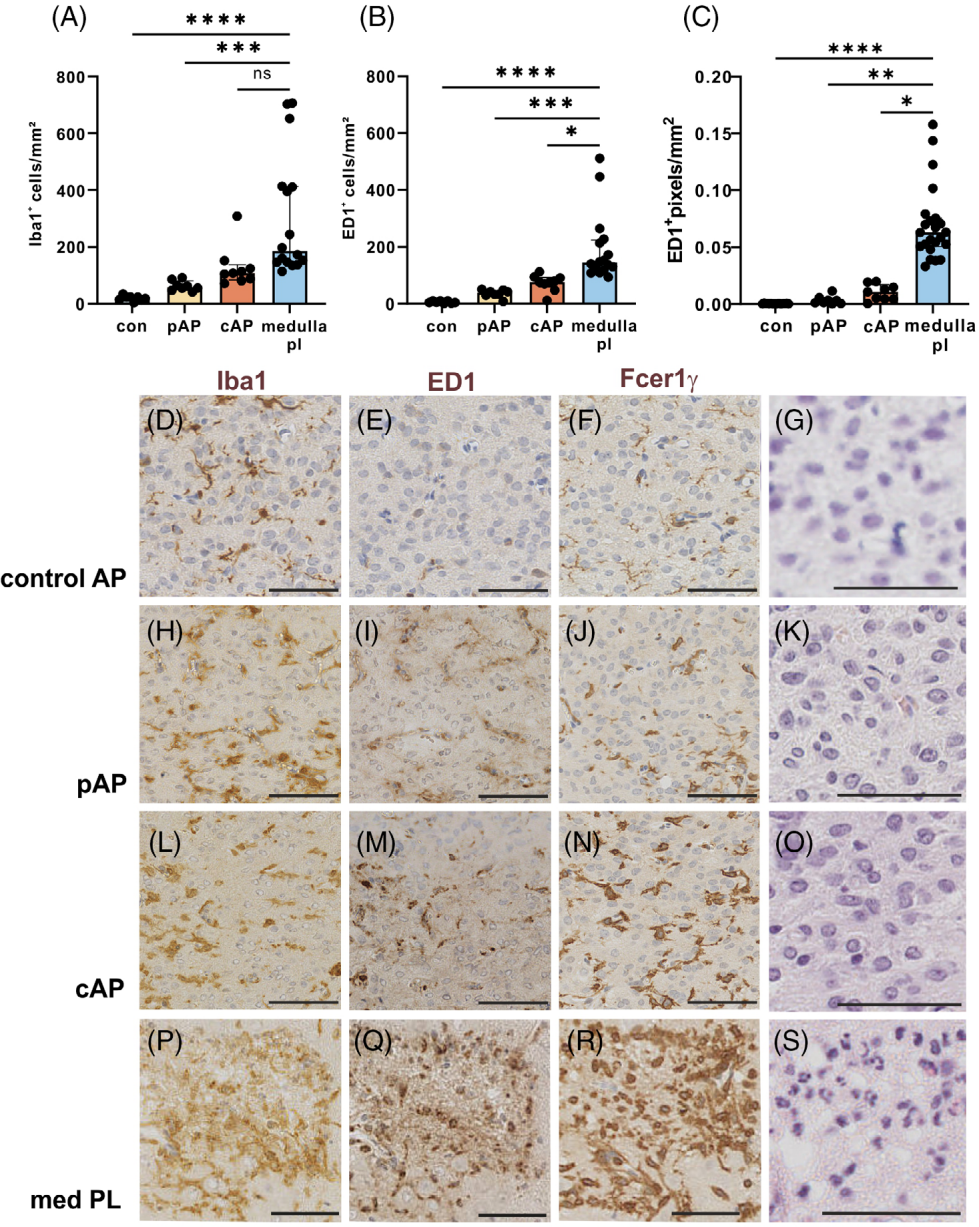
Activation of microglia/macrophages in the area postrema of control animals (con) or animals with patchy (pAP) or complete (cAP) loss of AQP4 reactivity, and in perivascular lesions with AQP4 loss in the medulla (medulla pl). (A, B) numbers of Iba1+ (A) and ED1+ (B) cells per mm^2^, and the numbers of ED1+ pixels (C) per mm^2^, using intact area postrema (*n* = 8 in A, B and *n* = 17 in C), area postrema with patchy loss of AQP4 reactivity (pAP, *n* = 8 in A–C), area postrema with complete loss of AQP4 reactivity (cAP, *n* = 9 in A–C), and medullary lesions with AQP4 loss (*n* = 16 in A, B and *n* = 23 in C). Data are presented as median with interquartile range. Statistical analysis was performed using Kruskal–Wallis test with the Bonferroni correction test. *p < 0.05, **p < 0.01, ***p < 0.001, ****p < 0.0001, n.s. not significant. (D–S) HE and immunohistochemical stainings of the control area postrema (D–G), of the area postrema with patchy (H–K) or complete loss (L–O) of AQP4 reactivity, and of a typical medullary perivascular lesion with AQP4 loss (P–S). The centers of the area postrema are shown. The sections were stained with antibodies specific for Iba1, with the ED1 antibody, and antibodies against Fcer1γ (for all these antibodies, reaction products were brown). Counterstaining was made with hematoxylin to show nuclei in blue. Bars = 50 μm in D–G, K, O–S, and 60 μm in H–J and L–N.

**FIGURE 7:**
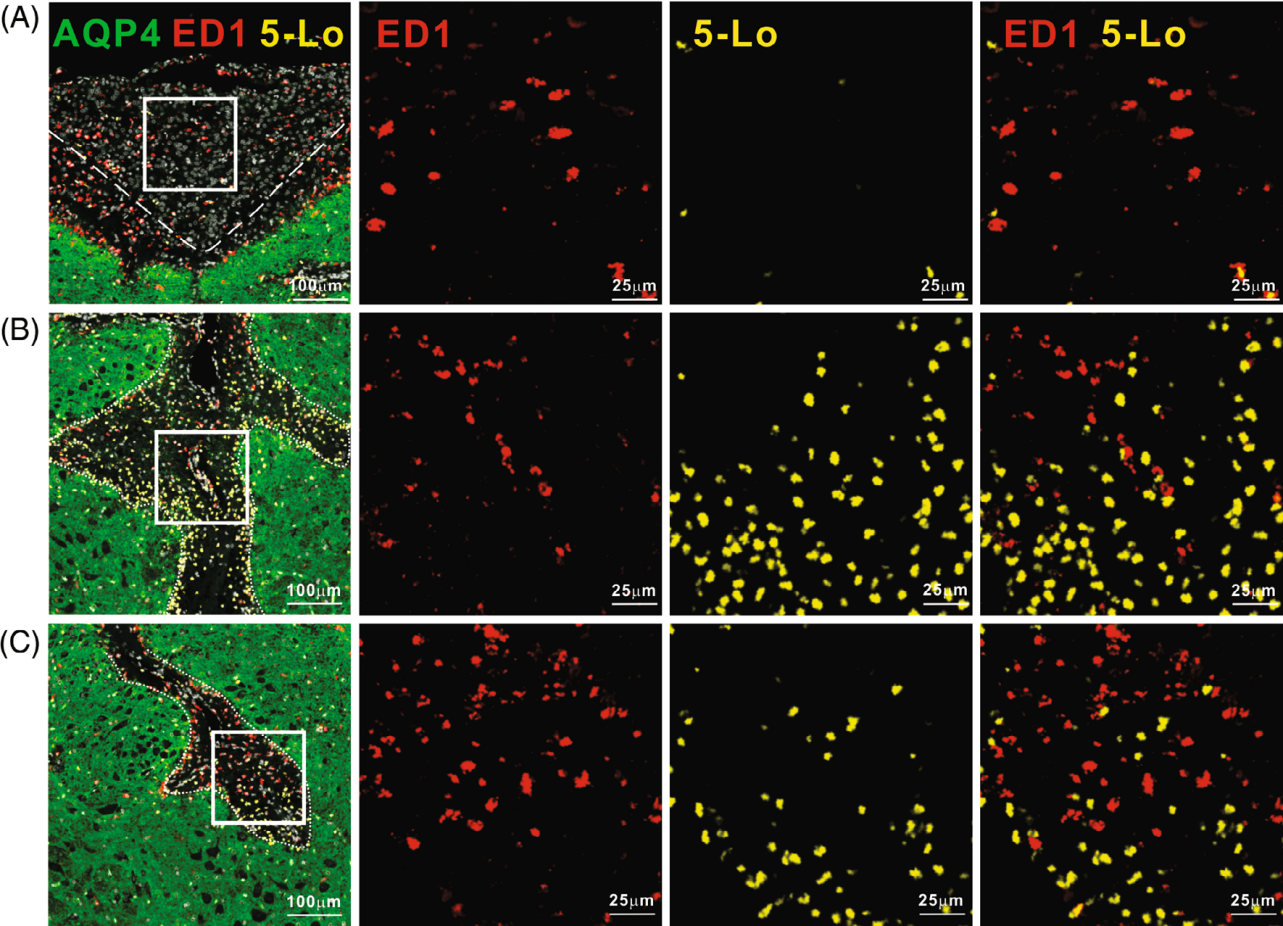
Stainings were made with AQP4 antibodies (green) to visualize astrocytes, with ED1 to identify macroglia/macrophages (red), and with antibodies against 5‐lipoygenase (5‐Lo) to detect neutrophils (yellow). The white broken line shows the border of the area postrema, the white dotted lines outline the perivascular lesions, and the white boxes show the tissue areas shown in higher magnification at the right side.

**FIGURE 8:**
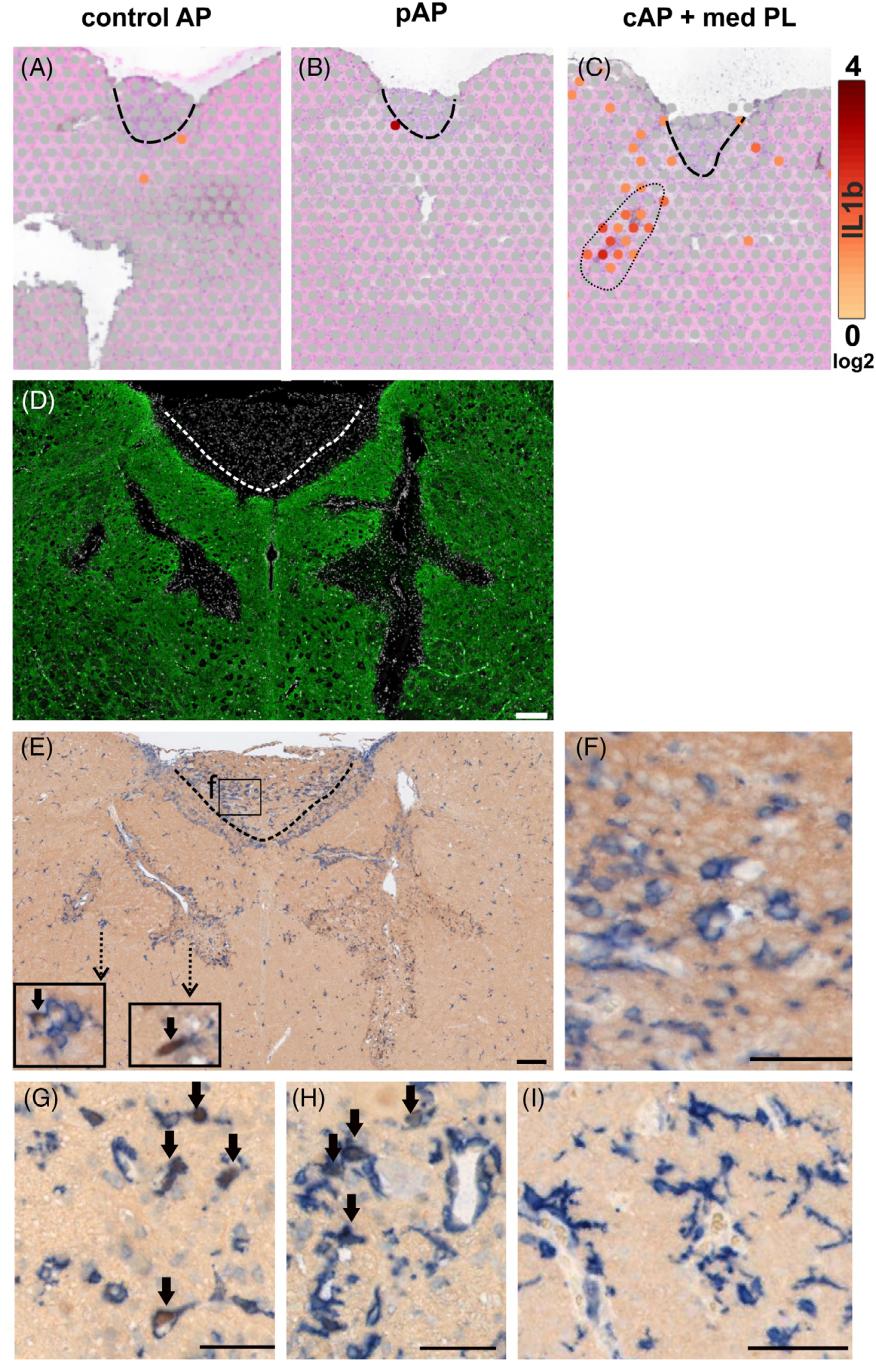
IL‐1β expression in the area postrema and perivascular lesions of the medulla. (A–C) Loupe browser images of interleukin1β (IL‐1β) transcripts in the intact area postrema (A), in the area postrema with patchy loss of AQP4 reactivity (B), and in the area postrema with complete loss of AQP4 reactivity and perivascular lesions in the medulla (C). The area postrema is outlined by broken lines, and the perivascular lesion by a dotted line. The color scale shows the log2 expression levels of the IL‐1β transcripts. (D–F) Staining of a medulla with complete loss of AQP4 reactivity in the area postrema (outlined by broken lines) and in inflammatory perivascular lesions in the medulla of a Lewis rat which was seropositive for AQP4‐abs for 48 h. Antibodies against AQP4 were used (positive reaction products green) to show the areas of AQP4 loss (section counterstained with DAPI to show nuclei (grey)) (D), and with antibodies against Iba1 (blue) and IL‐1β (brown) to reveal the distribution of Iba1^+^IL‐1β^+^ cells (E). The arrows with dotted lines in (E) point to a higher magnification of a small perivascular lesion (boxed) with an Iba1^+^IL‐1β^+^ cell (solid black arrow). (G–I) Iba1^+^IL‐1β^+^ cells (solid black arrows) in perivascular areas of the medulla (G, H), and absence of these cells from the area postrema (I) in a second Lewis rat, seropositive for AQP4‐abs for 96 h. The data shown here are representative for 4 animals with patchy loss of AQP4 reactivity in the area postrema (3 new cases, 1 archival case), 4 animals with complete AQP4 loss in the area postrema (4 new cases), and 2 animals with expanding perivascular lesions in the medulla. One additional animal had older medullary lesions which were essentially negative for Iba1^+^IL‐1β^+^ cells. Bars = 100 μm in D and E, and 50 μm in F–I.

In the control area postrema, few Iba1+ microglial cells were seen (Figure 6A) which were highly ramified (Figure 6D). ED1+ macrophages were rare (Figure 6B,E). Fcer1γ expression was found on ramified microglial cells (Figure 6F). In the area postrema with patchy (Figure 6H–K) and complete loss of AQP4 reactivity (Figure 6L–O), microglial cells became more numerous (Figure 6A,B) and were activated, as evidenced by the retraction and thickening of processes by Iba1+ (Figure 6H,L) and Fcer1γ+ cells (Figure 6J,N), and by the appearance of ED1+ cells (Figure 6I,M). In HE stainings, neutrophils could not be detected (Figure 6K,O). Medullary lesions with AQP4 loss displayed more Iba1+ (Figure 6A) than the AQP4 abs‐challenged area postrema. The Iba1+ cells were densely packed and had hardly discernable processes (Figure 6P). Medullary lesions also contained more ED1+ activated microglia/macrophages (Figure 6B) with an amoeboid phenotype (Figure 6Q), and had higher expression levels of ED1 (Figure 6C). These lesions also contained numerous Fcer1γ+ cells (Figure 6R) which represented activated microglia/macrophages and neutrophils. HE stainings and multiplex immunofluorescent labelling confirmed high numbers of neutrophils in medullary lesions (Figures 6S and 7). Transcripts for IL‐1β were absent in the area postrema (Figure 8A–C), but were readily detectable in perivascular lesions with AQP4 loss in the medulla (Figure 8C). This finding could be confirmed by immunohistochemistry (Figure 8D–I): IL‐1β expressing cells were essentially absent in the area postrema (Figure 8E,F,I), but were readily detectable at the rim of medullary lesions (Figure 8E,G,H).

We next analyzed the macrophage infiltration of the area postrema from NMOSD patients, compared to brain stem (BSL) and spinal cord lesions (SCL) of the same patients. Inflammatory cell infiltration differed markedly according to lesion stage in both BSL and SCL. CD68‐positive macrophage densities were higher in acute‐to‐subacute lesions than in chronic lesions (BSL: 934.1 ± 319.2/mm^2^ vs. 365.3 ± 224.6/mm^2^; SCL: 1265.2 ± 404.2/mm^2^ vs. 317.6 ± 166.5/mm^2^). Nevertheless, inflammatory infiltration in both lesion types was substantially greater than that observed in APL. To directly compare lesion severity within the same patients, paired analyses were performed in cases with both APL and corresponding SCL or BSL. In all paired cases, macrophage infiltration was consistently lower in APL than in corresponding SCL (Figure 2P) or BSL (Figure 2Q). Quantitatively, macrophage density was significantly lower in APL than in SCL (96.2 ± 66.7/mm^2^ vs. 429.6 ± 591.5/mm^2^, *p* = 0.01). A similar trend was also observed between APL and BSL (124.4 ± 88.8/mm^2^ vs. 677.0 ± 453.7/mm^2^, *p* = 0.13), although statistical significance was not reached because only three comparable cases were available for analysis.

Cumulatively, the results described above point to a dampened innate immune response in the AQP4‐antibody challenged area postrema.

## 4 DISCUSSION

Compared to other sites of the CNS, the area postrema has a remarkable resilience towards mAQP4‐abs induced tissue injury, although it shows evidence for minor neuroaxonal damage, as indicated by the upregulation of gene transcripts in the clusters “synapse pruning” and “response to axon injury,” which was noted in the area postrema with patchy loss of AQP4 reactivity and was possibly triggered by some axonal damage because of iono‐osmotic dysbalance caused by loss of AQP4 reactivity [27].

To identify the underlying mechanisms, we compared the pathological changes and the microglial/macrophage response between the AQP4‐abs challenged area postrema and lesions in the medulla, and used spatial transcriptomics together with functional annotation clustering to characterize the molecular and immunological landscape at these sites at unprecedented depth. Although we used a very strict cut‐off and did not consider transcripts with low average counts for functional annotation clustering, we observed profound differences in gene expression in response to mAQP4‐abs challenge between these two different sites, particularly affecting the inflammatory response:

Microgliosis was a key feature of the area postrema with patchy and complete loss of AQP4 reactivity and was evidenced by an increase in microglial cell numbers, by a retraction and thickening of microglial processes, and by the presence of ED1+ activated microglia/macrophages. The vast majority of these activated cells probably derived from local microglia because there was only very limited expression of chemokines in the AQP4‐abs challenged area postrema. Using our strict cut‐off criteria for analysis, only transcripts for Ccl2 and Ccl6 were upregulated. CCL2 and CCL6 are upregulated in astrocytes upon binding of AQP4‐abs [28], and both mediate microglial cell migration [29, 30], while CCL6 may additionally provide protection for neurons against glutamate neurotoxicity [31] and promote the transition of macrophages to the anti‐inflammatory M2 phenotype [32]. These effects might be particularly important in the absence of additional chemokines. Moreover, in the AQP4‐abs challenged area postrema of our experimental animals, 5‐LO+ granulocytes were essentially absent, the numbers of ED1+ activated microglial cells/macrophages was much lower than seen in the perivascular lesions, and IL1‐β expression was not detectable. Cumulatively, these findings are compatible with a dampened innate immune response in the area postrema.

In marked contrast, vasculocentric lesions originating in the medullary parenchyma displayed an acute inflammatory response, characterized by the presence of large numbers of 5‐LO+ granulocytes and ED1+ macrophages/activated microglial cells with an amoeboid phenotype. The majority of these ED1+ cells probably represented macrophages recruited from the periphery, because these medullary lesions were characterized by the production of transcripts for many different chemokines like Ccl2, Ccl3, Ccl6, Ccl7, Ccl9, Ccl24, Cxcl10, and Cxcl16 engaged in the chemotaxis of granulocytes, monocytes, and lymphocytes. The perivascular lesions were also characterized by IL1‐β producing cells. Hence, the cellular sources for the chemokines observed in these medullary lesions could be both AQP4‐abs binding astrocytes (for CCL2, CCL3, CCL6, and CCL7 [28]), and IL‐1β activated vascular endothelial cells [33]. Interestingly, IL1‐β protein production has been described in Iba1+ microglia/macrophages in perivascular spinal cord lesions of NMOSD patients [34].

The pivotal difference between the area postrema and the medullary parenchyma was the expression of Aldh1a2 in the area postrema, and its absence from the medullary parenchyma. Aldh1a2 is the key enzyme for the synthesis of all‐trans retinoic acid (ATRA) [35], a molecule with profound actions in the development and maintenance of the nervous system, and in neuronal differentiation [36]. Hence, it is not surprising that we also detected many upregulated genes in the intact area postrema which were involved in “brain development,” “neuron projection development,” “neuron differentiation,” and “neuroblast proliferation.” Retinoic acid synthesized by Aldh1a2 expressing cells is released and acts on neighboring cells, either promoting self‐renewal of stem cells or inducing processes of differentiation (depending on the cell‐type) and tissue regeneration [37]. Jointly, the findings described above are in line with the presence of a stem cell niche in the area postrema serving as a source of new neurons and glial cells under homeostatic conditions [38]. Interestingly, ATRA is not only important for development of, and differentiation in the nervous system [36], but also for immune processes in vivo and in vitro: It can prevent the activation of astrocytes and microglia, as seen in a rat model of cerebral ischemia [39], and can reduce the production of cytokine/chemokine transcripts and the release of cytokine/chemokine proteins in response to inflammatory stimulation in astrocytes [40, 41], but also in non‐astrocytic cells like adipocytes by inhibiting NF‐κB signaling [42]. ATRA also promotes an M1‐ to M2‐transition in macrophages [43] and alleviates the expression of IL‐1β in macrophages [44] and microglial cells [39]. Absence or very low concentrations of IL‐1β could translate to less activation of, and less chemokine production by vascular brain endothelial cells [33]. Further, ATRA may also specifically prevent the TNF‐α‐induced upregulation of the vascular cell adhesion molecule‐1, as shown for dermal microvascular endothelial cells in vitro [45], and may modulate the N‐glycan composition of intercellular adhesion molecule‐1, which was shown to inhibit cell adhesion to, and trans‐endothelial migration of pro‐monocytic U937 cells through human umbilicord vascular endothelial cell (HUVEC) monolayers [46]. And finally, ATRA may also inhibit the expression and activity of matrix metalloproteinase‐9 (MMP‐9) [47, 48], a key molecule for the migration of neutrophils across the basement membrane [49], and for the migration of macrophages during the inflammatory response [50]. Hence, the effects of ATRA in the area postrema could lower the amount of chemokine expression by local endothelial cells and astrocytes, interfere with adhesion of cells from the immune system to and their transmigration of endothelial cell barriers, and inhibit the migration of neutrophils and macrophages to this site. During homeostasis, the effects of ATRA could protect the area postrema which does not have a conventional blood–brain barrier from deleterious actions of blood‐borne immune cells and molecules. In the AQP4‐abs challenged area postrema, the action of ATRA might then guarantee that the immune response is dominated by local microglia/macrophages polarized towards an M2 phenotype rather than by cells recruited from outside the CNS, and that these cells have an anti‐inflammatory phenotype.

The specific and strong expression of Aldh1a2 in the area postrema, but not in the medullary parenchyma could then explain why perivascular lesions outside the area postrema are characterized by massive infiltration by different cells from the immune system, and by profound tissue destruction. To unambiguously prove that Aldh1a2 is the key molecule for dampening immune responses in the area postrema, additional animal experiments are needed. It needs to be shown that transgenic overexpression of this molecule elsewhere in the CNS protects these sites from the formation of inflammatory lesions with AQP4 loss, and that a specific knock‐down of Aldh1a2 expression in the area postrema renders this site susceptible to the formation of tissue destructive lesions with AQP4 loss. Similarly, if retinoic acids are indeed responsible for the differences in the immune response to AQP4‐abs between the area postrema and other sites of the CNS, providing high concentrations of retinoic acid or its metabolic precursors to Lewis rats challenged with the AQP4‐ab E5415A should prevent the formation of inflammatory lesions in spinal cord or brain areas apart from the area postrema, while interfering with the binding of retinoic acid to its receptors in the area postrema should abolish the protective effect of retinoic acids at this site, and should allow the formation of highly tissue destructive lesions at this site as well.

Interfering with retinoic acid levels could also have a major therapeutic impact for NMOSD patients. It has already been shown that an increase in serum‐retinol reduces the risk for new inflammatory CNS lesions in patients with relapsing–remitting multiple sclerosis [51] and animals with experimental autoimmune encephalomyelitis [52, 53, 54]. Although the resulting effects are mostly because of the influence of retinoic acids on the immune system rather than to effects on cells within the CNS, this treatment strategy could represent an attractive therapeutic regimen for relapse prevention in NMOSD patients as well. Application of synthetic retinoids or isotretinoin (13‐cis‐retinoic acid) at the time of antibody entry into the CNS might decrease tissue injury during the acute phase of NMOSD, because of the effects of retinoic acid on the protection of neurons [55, 56] and endothelial cells [57]. And given the positive effects of these molecules on neuronal regeneration [58], blood–brain barrier development [59], and remyelination [60], retinoic acids could even provide an attractive tool to support tissue repair.

## AUTHOR CONTRIBUTIONS

QY and YT analyzed and interpreted the data, and wrote the manuscript. QY performed spatial transcriptomic analysis, induced experimental NMOSD models, performed the immunohistochemical analysis of rat tissues, multiplex immunofluorescent labelling, and data analysis. YT made spatial transcriptomic analysis, the immunohistochemical analysis of human tissue, and data analysis. NY performed the MRI study. SB and MR purified the E5415A antibody, KMM helped with multiplex immunofluorescent labelling and data analysis. TB retrieved the published raw data from the rat area postrema, MEK made the cryosections of the medulla for spatial transcriptomics, IA and RH helped with the interpretation of data, and JB made immunohistochemical stainings of rat cryosections. MB designed the study, supervised the work, analyzed and interpreted data, and wrote the manuscript. All authors reviewed the manuscript critically for important intellectual content and approved the final version of the manuscript.

## FUNDING INFORMATION

This study was supported by the Austrian Science Fund (FWF grant 10.55776/PAT6054424 to MB; FWF grant I6565‐B, SYNABSII to RH, FWF grant 10.55776/EFP9 to IA, and FWF grant PIN9333824 to JB), Austrian Research Promotion Agency (FFG, project number FO999920011), and China Scholarship Council to QY (CSC 202306170046). MR and SB were supported by the intramural funding program of the Medical University Innsbruck Ph.D. Research Training Groups, Project 2022‐1‐2 “CONNECT.”

## CONFLICT OF INTEREST STATEMENT

The authors declare no conflicts of interest.

## Supporting information


**Figure S1.** Histological comparison of lesions in the medulla and in the spinal cord of an animal with complete loss of AQP4 reactivity in the area postrema and additional inflammatory lesions along the entire neuraxis. The reaction products of C9neo are shown in red, all other antibody reaction products (AQP4, mIgG, ED1, and GFAP) are shown in brown. The sections were counterstained with hematoxylin to show nuclei in blue. Note that both in the medulla and in the spinal cord, lesions are characterized by loss of AQP4 reactivity and the absence of GFAP reactivity indicating astrocyte destruction, by the presence of numerous CD68‐positive macrophages, and by the deposition of mIgG and C9neo.
**Figure S2.** (A) Single‐nuclei RNA sequencing of the rat area postrema and nucleus tractus solitarius identified clusters of cells with similar transcript expression, which are presented here as a uniform manifold approximation and projection (UMAP) dimension reduction plot of all nuclei color coded by cluster. Known marker genes for different cellular subtypes were then used to define these clusters on the cellular level. (B) Plot of marker genes used for the identification of cellular subtypes. The size of the dots is proportional to the percentage of cells expressing the gene, and the red‐scale of the dots indicates the average expression levels of the gene.
**Figure S3.** Regional distribution of Aldh1a2 immunoreactivity in the rat CNS. (A–D) Representative brightfield images showing Aldh1a2 staining in different CNS regions from one slide. Symbols indicate the manually selected regions of interest (ROIs) used for DAB optical density (OD) measurements, including the area postrema (AP), medulla, pons, cerebellum, cerebral brain, and spinal cord. The approximate boundary of the AP is indicated by a dashed outline. The dotted outline in (C) marks an area lacking DAB deposition because of a technical artifact; this area was not used for quantification. The red symbol in (D) indicates an ROI selected from the spinal cord grey matter. (E) Quantification of mean DAB OD in the selected ROIs shown in panels (A–D). Each point represents one manually selected ROI. Because of the small size of the AP, the entire AP region was measured as a single ROI. (F) Quantification of mean DAB OD in the AP and surrounding medulla. For each section, the entire AP was measured as one ROI because of its small size, while two ROIs were selected from the surrounding medulla. Matching symbol shapes indicate measurements from the same section. DAB OD was measured in QuPath using the default H‐DAB color deconvolution settings and the same analysis workflow for all ROIs. OD, optical density. Scale bars are indicated in the figure.
**Figure S4.** ALDH1A2 immunoreactivity in the human medulla across different rostrocaudal levels. (A–D) Upper and middle medullary levels, where the fourth ventricle remained widely open. Only minimal scattered ALDH1A2 immunoreactivity was observed near the ependymal surface of the fourth ventricle. (E–J) Lower medullary levels, where the fourth ventricle narrowed toward the obex. Distinct ALDH1A2‐positive structures became detectable in restricted dorsal medullary regions corresponding anatomically to the area postrema. (K, L) More caudal medullary level near the central canal transition, where the ALDH1A2‐positive structure became smaller and centrally localized. (M, N) Similar localization patterns observed in an ALS case. Boxed areas in the low‐magnification images are shown at higher magnification in the adjacent panels.
**Figure S5.** ALDH1A2 immunoreactivity in NMOSD area postrema lesions across different disease stages. (A–D) Case 1, representing acute‐stage NMOSD. (E–H) Case 3, representing subacute‐stage NMOSD. Areas of AQP4 loss (A, B, E, F) extended slightly beyond the ALDH1A2‐positive areas (C, D, G, H). (I–L) Case 6, representing chronic‐stage NMOSD. AQP4 loss was largely confined to the ALDH1A2‐positive area. (M–P) Area postrema tissue from an MS case. AQP4 immunoreactivity was preserved (M, N), and ALDH1A2 immunoreactivity was comparable to that observed in normal control tissues (O, P). Boxed areas in the low magnification images are shown at higher magnification in the adjacent panels.

**Table S1A** Functionally annotated charts for the GOTERM “Biological Processes” generated by DAVID for upregulated genes from the intact rat area postrema (control).

**Table S1B.** Functionally annotated charts for the GOTERM “Biological Processes” generated by DAVID for downregulated genes from the intact rat area postrema (control).

**Table S2A.** Functionally annotated charts for the GOTERM “Biological Processes” generated by DAVID for upregulated genes from the rat area postrema with patchy AQP4 loss.

**Table S2B.** Functionally annotated charts for the GOTERM “Biological Processes” generated by DAVID for downregulated genes from the rat area postrema with patchy AQP4 loss.

**Table S3A.** Functionally annotated charts for the GOTERM “Biological Processes” generated by DAVID for upregulated genes from the rat area postrema with complete AQP4 loss.

**Table S3B.** Functionally annotated charts for the GOTERM “Biological Processes” generated by DAVID for downregulated genes from the rat area postrema with complete AQP4 loss.

**Table S4A.** Functionally annotated charts for the GOTERM “Biological Processes” generated by DAVID for upregulated genes from the perivascular lesion in the rat medulla.

**Table S4B.** Functionally annotated charts for the GOTERM “Biological Processes” generated by DAVID for downregulated genes from the perivascular lesion in the rat medulla.

## Data Availability

The data that support the findings of this study are available in the Supporting Information of this article. The fastq files of the sequence reads used for spatial transcriptomics were deposited at the sequence read archive (SRA) database and can be accessed as follows: For the medulla/area postrema of the control Lewis rat (0 days seropositive for AQP4‐abs, animal A): NCBI/SRA accession numbers PRJNA1258753 and PRJNA1262739; of the Lewis rat seropositive for AQP4‐abs for 24 h, animal B: PRJNA1262139 and PRJNA1262895; and of the Lewis rat seropositive for AQP4‐abs for 48 h, animal C: PRJNA1262368 and PRJNA1263155.
