# Supplementary material for: The immunological landscape of the area postrema in neuromyelitis optica spectrum disorders
Source: Brain Pathol. 2026 Jul 26:e70127. Online ahead of print. doi: 10.1111/bpa.70127 (PMC13402650; doi:10.1111/bpa.70127)
Supplement: Supplementary file 1 — Figure S1. Histological comparison of lesions in the medulla and in the spinal cord of an animal with complete loss of AQP4 reactivity in the area postrema and additional inflammatory lesions along the entire neuraxis. The reaction products of C9neo are shown in red, all other antibody reaction products (AQP4, mIgG, ED1, and GFAP) are shown in brown. The sections were counterstained with hematoxylin to show nuclei in blue. Note that both in the medulla and in the spinal cord, lesions are characterized by loss of AQP4 reactivity and the absence of GFAP reactivity indicating astrocyte destruction, by the presence of numerous CD68‐positive macrophages, and by the deposition of mIgG and C9neo. Figure S2. (A) Single‐nuclei RNA sequencing of the rat area postrema and nucleus tractus solitarius identified clusters of cells with similar transcript expression, which are presented here as a uniform manifold approximation and projection (UMAP) dimension reduction plot of all nuclei color coded by cluster. Known marker genes for different cellular subtypes were then used to define these clusters on the cellular level. (B) Plot of marker genes used for the identification of cellular subtypes. The size of the dots is proportional to the percentage of cells expressing the gene, and the red‐scale of the dots indicates the average expression levels of the gene. Figure S3. Regional distribution of Aldh1a2 immunoreactivity in the rat CNS. (A–D) Representative brightfield images showing Aldh1a2 staining in different CNS regions from one slide. Symbols indicate the manually selected regions of interest (ROIs) used for DAB optical density (OD) measurements, including the area postrema (AP), medulla, pons, cerebellum, cerebral brain, and spinal cord. The approximate boundary of the AP is indicated by a dashed outline. The dotted outline in (C) marks an area lacking DAB deposition because of a technical artifact; this area was not used for quantification. The red symbol in (D) indicates an [file BPA-9999-e70127-s002.docx]

**The Immunological Landscape of the Area Postrema in Neuromyelitis Optica Spectrum Disorders**

^1^*Qian Yu, ^2^*Yoshiki Takai, ^2^Naoya Yamazaki, ^3^Sarah Brandl, ^1^Katharina M. Mair, ^1^Thibault Bouderlique, ^1^Maria Eleni Kastriti, ^1^Igor Adameyko, ^4^Romana Höftberger, ^3^Markus Reindl, ^1^Jan Bauer, ^1^Monika Bradl

^1^Medical University Vienna, Center for Brain Research, Division of Neuroimmunology, Vienna, Austria; ^2^Department of Neurology, Tohoku University Graduate School of Medicine, Sendai, Japan;

^3^Medical University of Innsbruck, Clinical Department of Neurology, Innsbruck, Austria; ^4^Medical University of Vienna, Department of Neurology, Division of Neuropathology and Neurochemistry, Vienna, Austria

*both authors contributed equally to this study

Short running title: Immunological landscape of area postrema in NMOSD

**SUPPLEMENTARY INFORMATION**

**
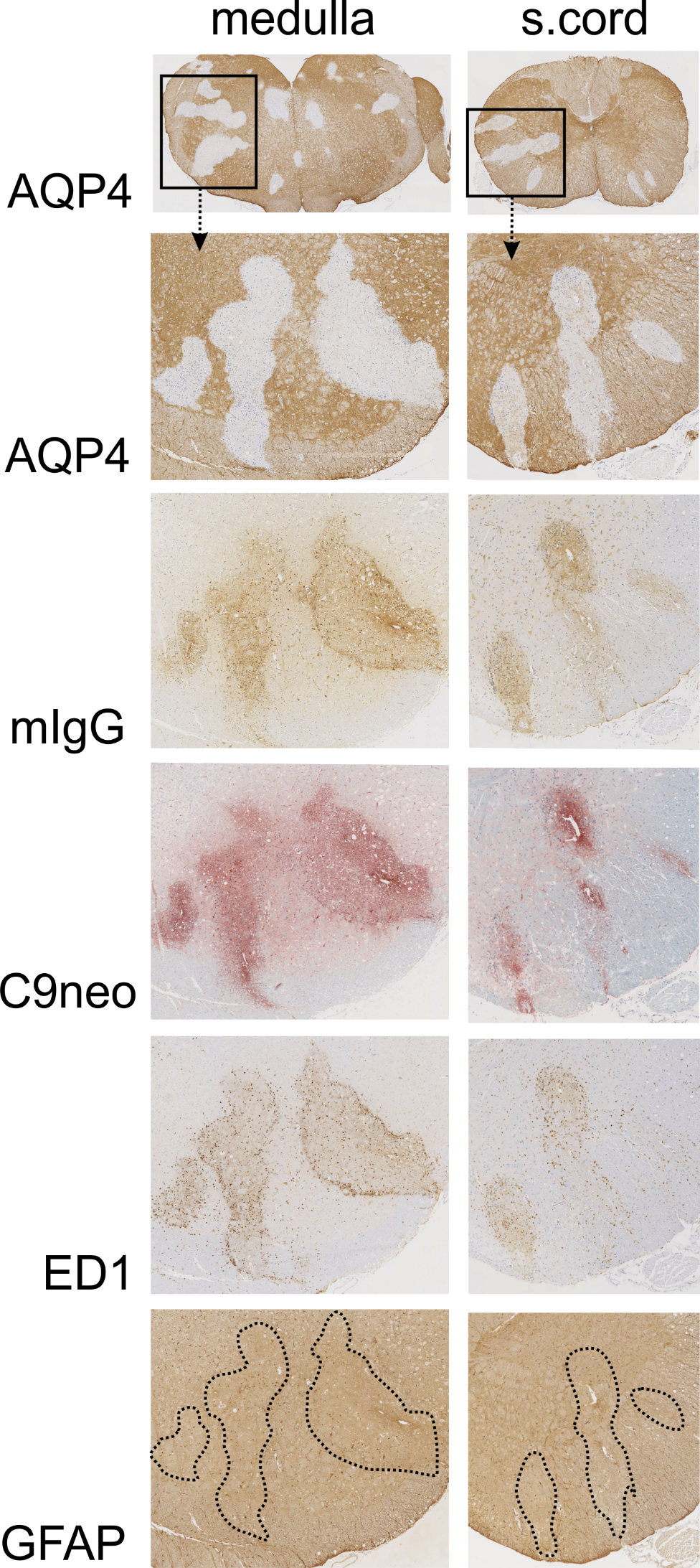
**

**Suppl fig 1**

Histological comparison of lesions in the medulla and in the spinal cord of an animal with complete loss of AQP4 reactivity in the area postrema and additional inflammatory lesions along the entire neuraxis. The reaction products of C9neo are shown in red, all other antibody reaction products (AQP4, mIgG, ED1, and GFAP) are shown in brown. The sections were counterstained with hematoxylin to show nuclei in blue.

Note that both in the medulla and in the spinal cord, lesions are characterized by loss of AQP4 reactivity and the absence of GFAP reactivity indicating astrocyte destruction, by the presence of numerous CD68-positive macrophages, and by the deposition of mIgG and C9neo.

**
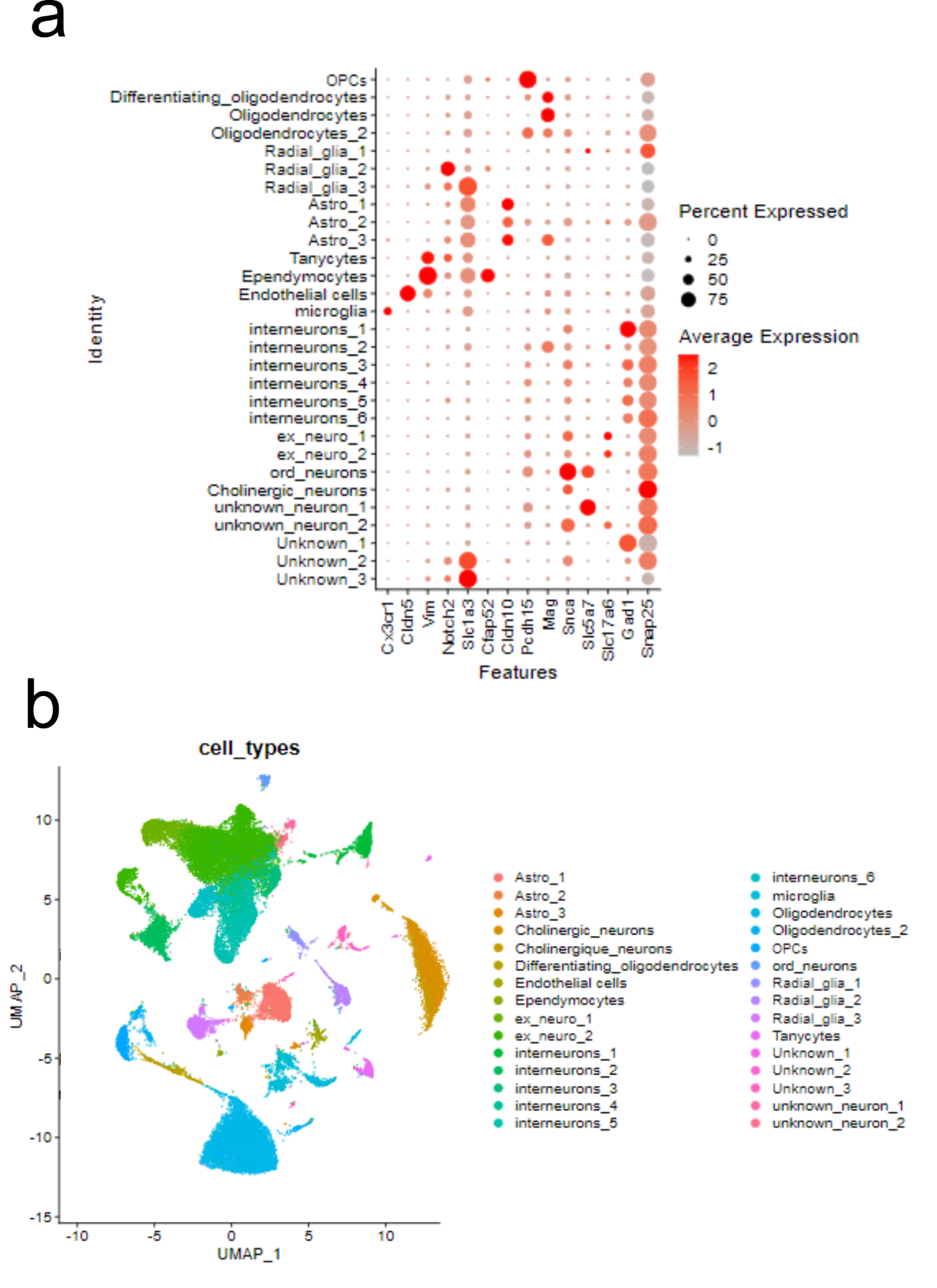
**

**Suppl fig 2**

(a) single nuclei RNA sequencing of the rat area postrema and nucleus tractus solitarius identified clusters of cells with similar transcript expression, which are presented here as a uniform manifold approximation and projection (UMAP) dimension reduction plot of all nuclei color coded by cluster. Known marker genes for different cellular subtypes were then used to define these clusters on the cellular level.

(b) Plot of marker genes used for the identification of cellular subtypes. The size of the dots is proportional to the percentage of cells expressing the gene, and the red-scale of the dots indicates the average expression levels of the gene.


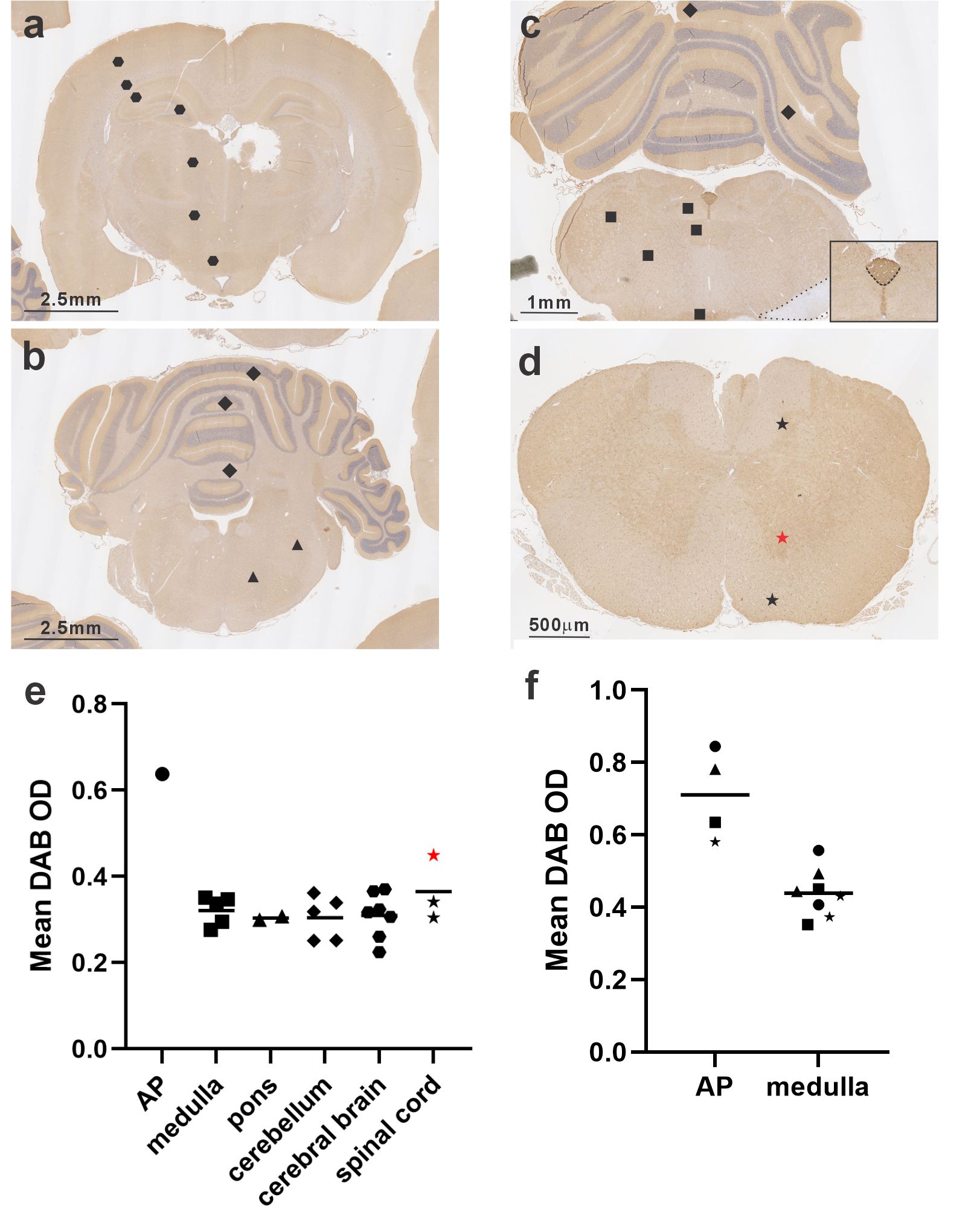


**Suppl. fig. 3**

Regional distribution of Aldh1a2 immunoreactivity in the rat CNS.

a–d, Representative brightfield images showing Aldh1a2 staining in different CNS regions from one slide. Symbols indicate the manually selected regions of interest (ROIs) used for DAB optical density (OD) measurements, including the area postrema (AP), medulla, pons, cerebellum, cerebral brain, and spinal cord. The approximate boundary of the AP is indicated by a dashed outline. The dotted outline in fig.c marks an area lacking DAB deposition due to a technical artifact; this area was not used for quantification. The red symbol in fig.d indicates an ROI selected from the spinal cord grey matter.

e, Quantification of mean DAB OD in the selected ROIs shown in panels a–d. Each point represents one manually selected ROI. Due to the small size of the AP, the entire AP region was measured as a single ROI.

f, Quantification of mean DAB OD in the AP and surrounding medulla. For each section, the entire AP was measured as one ROI due to its small size, while two ROIs were selected from the surrounding medulla. Matching symbol shapes indicate measurements from the same section. DAB OD was measured in QuPath using the default H-DAB color deconvolution settings and the same analysis workflow for all ROIs.

OD, optical density. Scale bars are indicated in the figure.


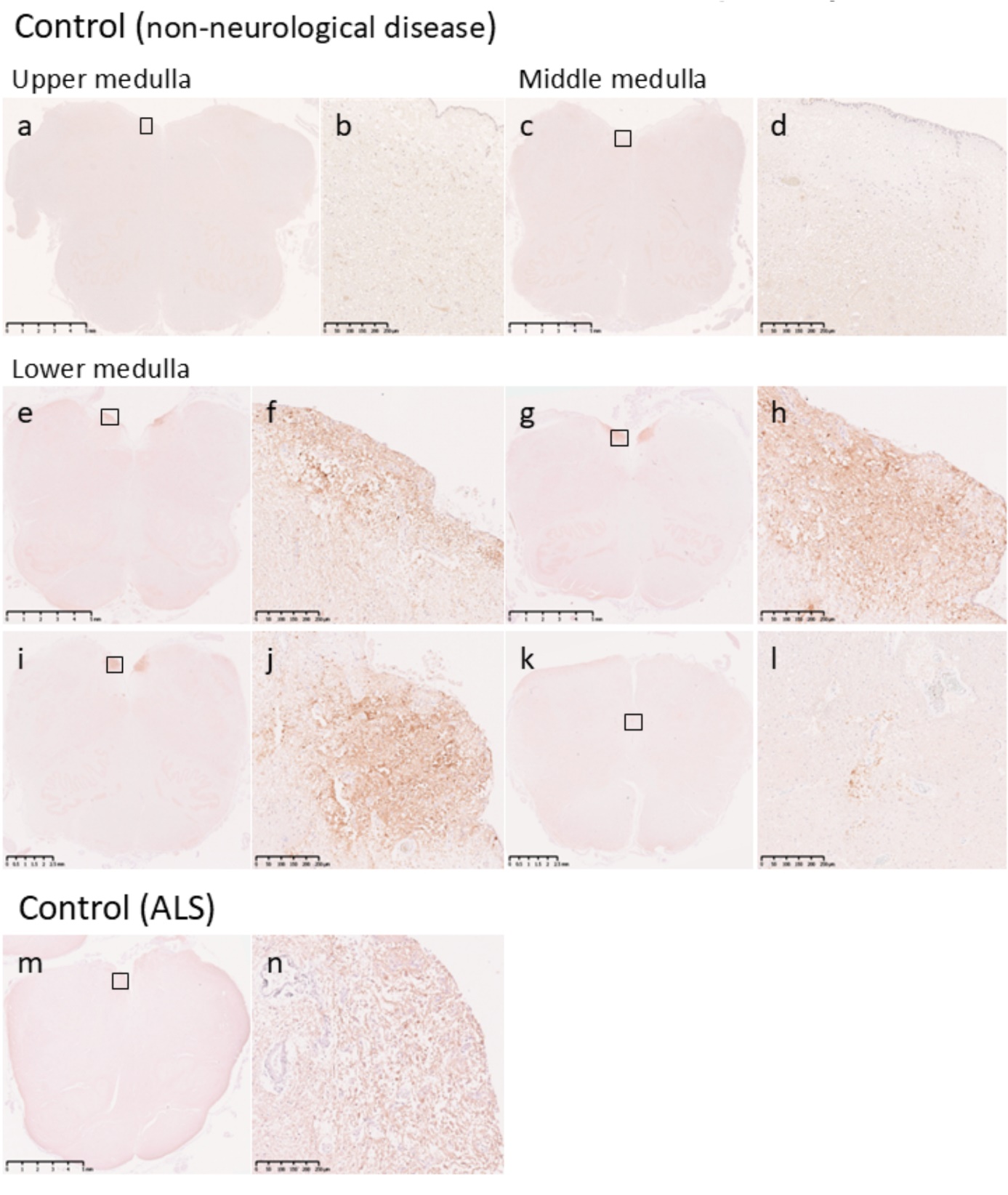


**Suppl fig. 4:**

ALDH1A2 immunoreactivity in the human medulla across different rostrocaudal levels.
(a–d) Upper and middle medullary levels, where the fourth ventricle remained widely open. Only minimal scattered ALDH1A2 immunoreactivity was observed near the ependymal surface of the fourth ventricle. (e–j) Lower medullary levels, where the fourth ventricle narrowed toward the obex. Distinct ALDH1A2-positive structures became detectable in restricted dorsal medullary regions corresponding anatomically to the area postrema. (k, l) More caudal medullary level near the central canal transition, where the ALDH1A2-positive structure became smaller and centrally localized. (m, n) Similar localization patterns observed in an ALS case. Boxed areas in the low-magnification images are shown at higher magnification in the adjacent panels.


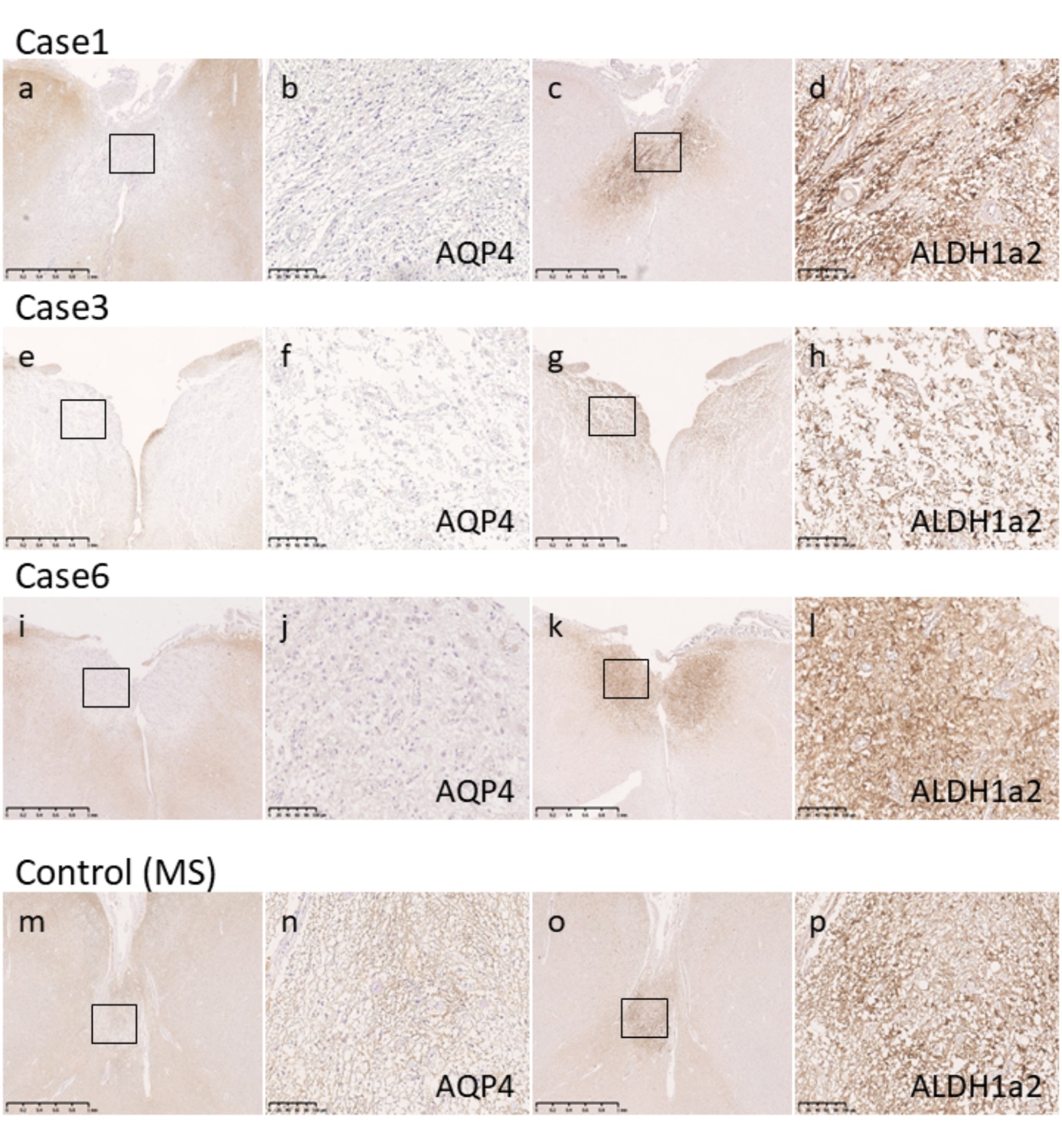


**Suppl fig. 5**

ALDH1A2 immunoreactivity in NMOSD area postrema lesions across different disease stages. (a–d) Case 1, representing acute-stage NMOSD. (e–h) Case 3, representing subacute-stage NMOSD. Areas of AQP4 loss (a, b, e, f) extended slightly beyond the ALDH1A2-positive areas (c, d, g, h). (i–l) Case 6, representing chronic-stage NMOSD. AQP4 loss was largely confined to the ALDH1A2-positive area. (m–p) Area postrema tissue from an MS case. AQP4 immunoreactivity was preserved (m, n), and ALDH1A2 immunoreactivity was comparable to that observed in normal control tissues (o, p). Boxed areas in the low magnification images are shown at higher magnification in the adjacent panels.

**Suppl Table 1**

a) Functionally annotated charts for the GOTERM “Biological Processes” generated by DAVID for upregulated genes from the intact rat area postrema (control).

b) Functionally annotated charts for the GOTERM “Biological Processes” generated by DAVID for downregulated genes from the intact rat area postrema (control).

**Suppl Table 2**

a) Functionally annotated charts for the GOTERM “Biological Processes” generated by DAVID for upregulated genes from the rat area postrema with patchy AQP4 loss.

b) Functionally annotated charts for the GOTERM “Biological Processes” generated by DAVID for downregulated genes from the rat area postrema with patchy AQP4 loss.

**Suppl Table 3**

a) Functionally annotated charts for the GOTERM “Biological Processes” generated by DAVID for upregulated genes from the rat area postrema with complete AQP4 loss.

b) Functionally annotated charts for the GOTERM “Biological Processes” generated by DAVID for downregulated genes from the rat area postrema with complete AQP4 loss.

**Suppl Table 4**

a) Functionally annotated charts for the GOTERM “Biological Processes” generated by DAVID for upregulated genes from the perivascular lesion in the rat medulla.

b) Functionally annotated charts for the GOTERM “Biological Processes” generated by DAVID for downregulated genes from the perivascular lesion in the rat medulla.
